# A dual catalytic architecture promotes C-2 stereoinversion of CDP–glucose by the CDP–tyvelose 2-epimerase from *Thermodesulfatator atlanticus*

**DOI:** 10.1016/j.jbc.2026.111384

**Published:** 2026-03-19

**Authors:** Christian Rapp, Stevie van Overtveldt, Pedro A. Sánchez-Murcia, Martin Pfeiffer, Koen Beerens, Magdalena Merkaš, Tea Pavkov-Keller, Tom Desmet, Bernd Nidetzky

**Affiliations:** 1 Institute of Biotechnology and Biochemical Engineering, Graz University of Technology, NAWI Graz, Graz, Austria; 2 Centre for Synthetic Biology, Department of Biotechnology, Ghent University, Ghent, Belgium; 3 Laboratory of Computer-Aided Molecular Design, Division of Medicinal Chemistry, Otto- Loewi Research Center, Medical University of Graz, BioTechMed-Graz, Graz, Austria; 4 Institute of Molecular Biotechnology, Graz University of Technology, NAWI Graz, Graz, Austria; 5 Institute of Molecular Biosciences, University of Graz, Graz, Austria; 6 Austrian Centre of Industrial Biotechnology (acib), Graz, Austria

**Keywords:** enzyme catalysis, enzyme mechanism, crystal structure, nucleotide, glucose, conformational change, protein conformation, substrate specificity

## Abstract

The CDP–tyvelose 2-epimerase from *Thermodesulfatator atlanticus* (*Ta*TyvE) catalyzes the C-2 epimerization of CDP–glucose to CDP–mannose. The enzyme uses NAD-dependent oxidation–reduction to achieve C-2 configurational inversion of the substrate. Here, we report the 2.60-Å crystal structure of tetrameric *Ta*TyvE with NAD^+^ bound in all subunits and CDP bound in one (Protein Data Bank code: 9RL0). Binding of CDP orders the Gly197–Trp207 loop, closing over the sugar binding pocket. The active site of the ternary complex is well preorganized, with only moderate induced-fit conformational changes. Molecular dynamics simulations and site-directed mutagenesis suggest that *Ta*TyvE employs a dual catalytic architecture to control substrate specificity. Asn125, within the TNK segment (Thr124–Asn125–Lys126), promotes sampling of catalytically plausible glucose conformations. The VAM segment (Val83–Ala84–Met85) permits broader conformational flexibility for mannose through backbone contacts. This is supported by a modest ∼12-fold activity reduction in the Q205A variant. In contrast, N125A abolishes activity. Substrate analogs featuring deoxygenation, stereoinversion, or fluorination at the C-4 were synthesized to examine the role of the sugar C4–OH. Simulations indicated that the C4–OH interacts with Val83, Asn125, and Gln205. Activity was lost with CDP–4-deoxy-glucose and minimally recovered with CDP–4-fluoro-glucose. Removing the C5-hydroxymethyl group to restrict substrate positioning flexibility enhanced the reaction rate. Overall, these results highlight the important interplay of structural preorganization and conformational flexibility in *Ta*TyvE for enzyme activity and specificity in the C-2 epimerization of CDP–glucose.

CDP–tyvelose 2-epimerase (TyvE) belongs to a distinct family (SDR1E (1); Enzyme Commission [EC] 5.1.3.10) of sugar nucleotide epimerases within the large short-chain dehydrogenase/reductase (SDR) superfamily of enzymes (2, 3, 4). TyvE catalyzes the C-2 epimerization of CDP–paratose (CDP–Par) to CDP–tyvelose (CDP–Tyv; Fig. 1*A*) in the microbial biosynthesis of 3,6-dideoxy-hexoses (5, 6, 7, 8, 9, 10). d-Paratose (Par; 3,6-dideoxy-d-glucose; systematic name: 3,6-dideoxy-d-*ribo*-hexose) and d-tyvelose (Tyv; 3,6-dideoxy-d-mannose; systematic name: 3,6-dideoxy-d-*arabino*-hexose) are naturally rare monosaccharides found in the *O*-antigen regions of the exopolysaccharide lipopolysaccharides of Enterobacteriaceae and other bacteria (11, 12). d-Tyvelose is a major immunological determinant of pathogens, such as *Yersinia pseudotuberculosis* and *Salmonella typhi*. PCR screening of the TyvE gene is therefore used for the detection of *Salmonella* spp. in the clinics as well as in food and environmental monitoring (13, 14, 15, 16).

**Figure 1:**
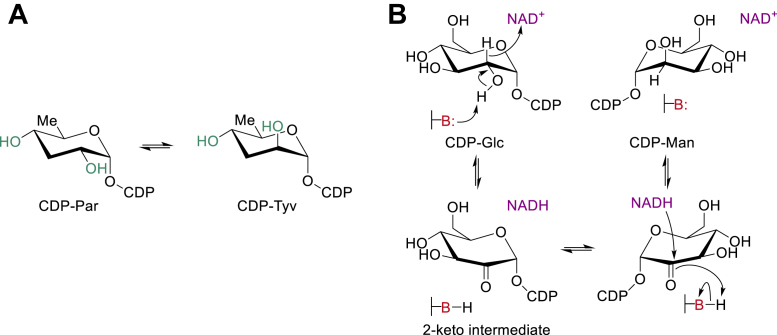
**Proposed reaction mechanism for TyvE.***A,* the biosynthetic reaction of TyvE, converting CDP–Par into CDP–Tyv. The hydroxyl groups at C-2 and C-4 (highlighted in *green*) are potential recognition sites for TyvE. *B,* C-2 epimerization by nicotinamide coenzyme–dependent oxidation and reduction *via* a transient 2-keto-intermediate. CDP–Par, CDP–paratose (CDP–3,6-dideoxy-α-d-glucose; CDP–Tyv, CDP–tyvelose (CDP–3,6-dideoxy-α-d-mannose); TyvE, CDP–tyvelose 2-epimerase.

TyvE from the anaerobic thermophile *Thermodesulfatator atlanticus* (*Ta*TyvE) has recently attracted attention for its activity with nondeoxygenated sugar substrates, converting nucleoside diphosphate–activated derivatives of d-glucose into the corresponding d-mannose compounds (17, 18). The particular specificity of *Ta*TyvE can be relevant in applied biocatalysis to provide nucleoside diphosphate–activated d-mannoses as glycosylation donors for oligosaccharide and glycoconjugate synthesis (19, 20, 21, 22).

Mechanistically, TyvE achieves configurational inversion of the substrate C-2 through a two-step oxidation–reduction reaction that proceeds *via* a transient CDP–2-keto-sugar intermediate (6, 23, 24). The substrate is oxidized by hydrogen abstraction to an NAD^+^ coenzyme that is tightly bound to the enzyme (Fig. 1*B*). A general base of the enzyme assists the reaction by catalytically removing the proton from the 2′-OH group (6, 23, 24). The tyrosine (Tyr164 of *Ta*TyvE) from the conserved SDR active site signature motif is the most probable candidate residue to fulfill that role (24). Free rotation of the 2-keto-hexopyranose moiety is required so that hydrogen-transfer reduction by enzyme–NADH occurs in a nonstereospecific fashion from either face of the carbonyl group, resulting in inversion of the stereochemistry at C-2 (6, 23, 24). The proposed TyvE reaction (Fig. 1*B*) has clear mechanistic analogies to the reactions of sugar nucleotide C-4 epimerases, such as UDP- or GDP-galactose 4-epimerase (GalE; (25, 26, 27, 28, 29)), UDP-*N*-acetylglucosamine 4-epimerase (GlcNAcE; (30, 31)), and UDP-glucuronic acid 4-epimerase (GlcAE; (32, 33, 34)). Like GalE, GlcNAcE, and GlcAE, TyvE handles the chemically challenging task of epimerizing a nonactivated carbon stereocenter of a carbohydrate substrate (6, 23, 24).

Epimerase reactions by oxidation and reduction confront a common conundrum, namely that conformational rigidity required for precise positioning in hydrogen transfer to and from the nicotinamide C-4 must be combined, and coordinated, with conformational flexibility required for rotatability of the transient ketointermediate (6, 24, 25, 27, 29, 34). The relationship between structural preorganization and conformational flexibility is an important current topic of mechanistic enzymology that receives high interest within the overall thematic framework of protein motions and enzyme catalysis (34, 35, 36, 37, 38, 39, 40, 41, 42). The picture emerging from a number of studies on the C-4 epimerases is that the binding pockets for NAD^+^ and sugar nucleotide substrate are structurally well preorganized, and the active site at the juncture of the two binding pockets is conformationally very rigid (25, 33, 34, 42, 43, 44). The general point is highlighted by the example of the GlcAE from *Bacillus cereus*: X-ray structures of enzyme–NAD^+^ in complex with UDP-glucuronic acid or UDP-galacturonic acid differ only in the orientation of a single peptide bond, that between residues Pro85 and Gly86, as shown in Figure S1 (33). The substrate complex involves interaction of the main-chain oxygen of Pro85 with the sugar 3-OH of UDP-glucuronic acid (33). In the flipped orientation of the product complex, the main-chain nitrogen of Gly86 interacts with the 5-carboxylate group of UDP-galacturonic acid (33). The current study of *Ta*TyvE was performed with the aim of characterizing the important interplay of structural preorganization and flexibility in the C-2 epimerization of CDP–glucose.

Here, we report the 2.60 Å crystal structure of *Ta*TyvE (Protein Data Bank [PDB] ID: 9RL0). The enzyme crystallizes as a dimer of dimers, with each dimer subunit containing tightly bound NAD^+^, but only one subunit had CDP bound additionally. Subunit structural comparison showed that the loop of residues Gly197 to Trp207 was mainly disordered in the subunit with bound NAD^+^ but became ordered upon CDP binding. We show that the induced-fit loop ordering in *Ta*TyvE contributed to the formation of a structurally confined binding pocket for the hexose moiety of the CDP–substrate.

Molecular dynamics (MD) simulations, site-directed mutagenesis, and synthesis of CDP–glucose/CDP–mannose (CDP–Man) analogs reveal that *Ta*TyvE employs a dual catalytic architecture to control substrate specificity. Asn125 in the TNK segment orients glucose in a rigid ^4^*C*_1_ chair conformation, whereas the VAM segment enables flexible mannose binding through backbone interactions. Overall, we present evidence in support of the suggestion that enzymatic C-2 epimerization of CDP–glucose requires only a small degree of enzyme conformational flexibility. Parsimony of conformational change associated with torsional motions of the ketointermediate appears to be a common defining feature of catalysis by sugar nucleotide epimerases of the SDR superfamily type (25, 33, 34, 43, 44).

## Results

### *Ta*TyvE crystal structure

**Figure 2:**
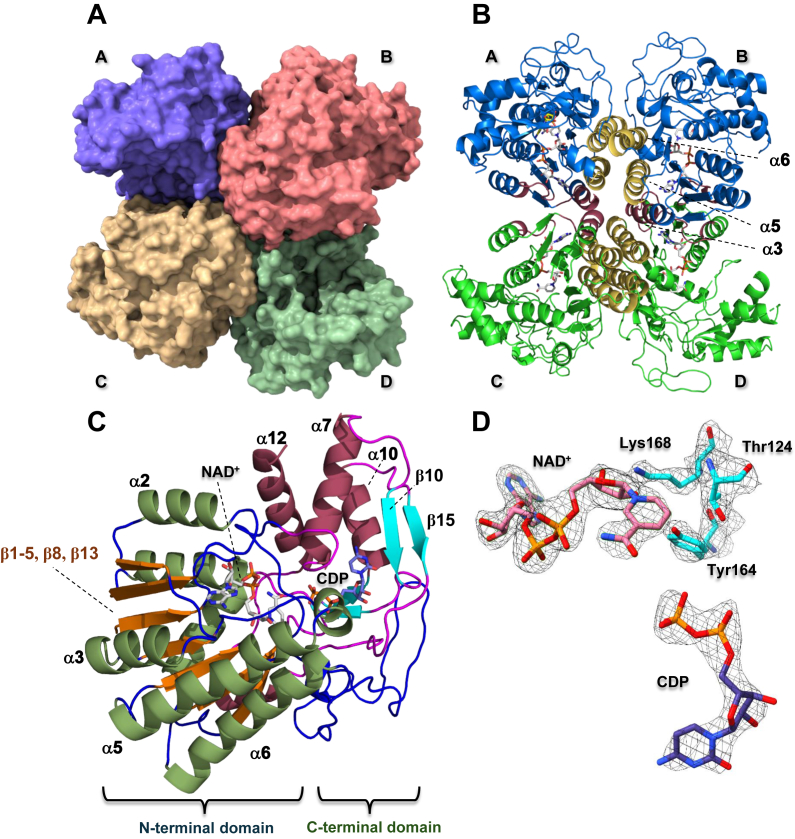
**The *Ta*TyvE crystal structure with bound NAD^+^ and CDP at 2.60 Å resolution.** (*A*) *surface* and (*B*) *cartoon* representation of the tetrameric assembly of *Ta*TyvE protomers A–D in the crystal to form a dimer of A/B and C/D dimer pairs. The α-helices of the four-helix bundle dimer interface are shown in *dark yellow*. The protomer regions participating in dimer–dimer interactions are shown in *dark violet*. *C,* the *Ta*TyvE protomer is comprised of two lobes: the N-terminal Rossmann-fold domain (α-helices: *dark green*; β-strands: *orange*; and loops: *blue*) for NAD^+^ binding and the C-terminal domain (α-helices: *dark violet*; β-strands: *cyan*; and loops: *magenta*) for CDP-substrate binding are highlighted by color (see Fig. 3*B* for details on secondary structural motifs). The NAD^+^ ligand is shown in *gray*, and the CDP ligand is shown in *blue*. *D,* 2*F*_*o*_ - *F*_*c*_ electron density map contoured at 1σ showing the SDR catalytic triad (Tyr164, Thr124, and Lys168), CDP, and NAD^+^ (labeled). SDR, short-chain dehydrogenase/reductase; *Ta*TyvE, CDP–tyvelose 2-epimerase from *Thermodesulfatator atlanticus*.

**Figure 3:**
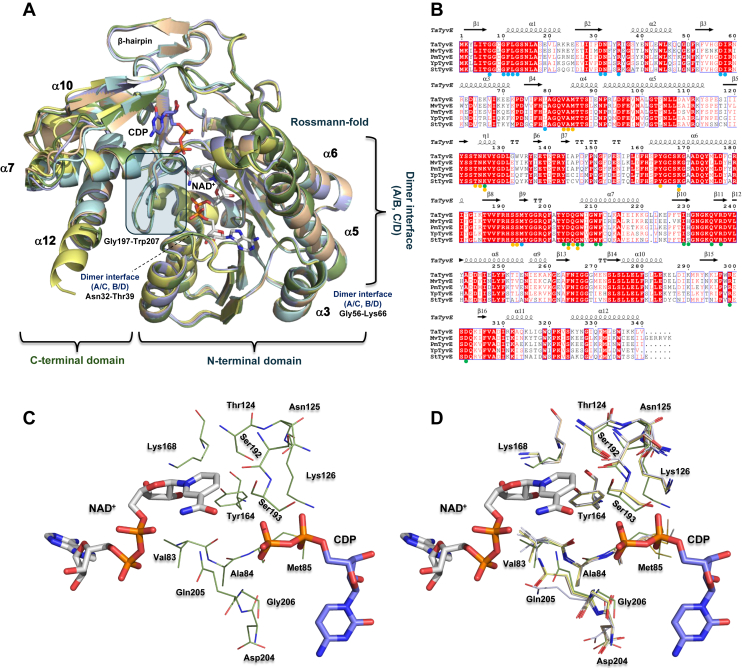
**Superposition and sequence alignment of characterized TyvEs with secondary structural motifs.***A,* structural superposition of *Ta*TyvE (*green*; *this work*), *St*TyvE (*pale cyan*; TyvE from *Salmonella typhi*; UniProt ID: P14169; PDB ID: 1ORR; (23)), *Mv*TyvE (*pale yellow*; TyvE from *Methanocaldococcus villosus*; UniProt ID: N6VPH4; AlphaFoldDB: AF-N6VPH4-F1-v6; (47)), *Pm*TyvE (*pale blue*; TyvE from *Persephonella marina*; UniProt ID: C0QSV4; AlphaFoldDB: AF-C0QSV4-F1-v6; (47)), and *Yp*TyvE (*pale brown*; TyvE from *Yersinia pseudotuberculosis*; UniProt ID: Q56944; AlphaFoldDB: AF-Q56944-F1-v6; (6)). *B,* sequence alignment of TyvEs with conserved secondary structural elements. Helices (α1–α12) and strands are labeled (β1–β16); η, η-helix; T, turn. Dots below the alignment mark binding sites for CDP (*green dots*), NAD^+^ (*blue dots*), and the sugar moiety (*orange dots*). Alignments were generated using ESPript (83). (*C*) *Ta*TyvE sugar binding pocket and (*D*) structural overlay with characterized TyvEs as shown in *A*. *Mv*TyvE, CDP–tyvelose 2-epimerase from *Methanocaldococcus villosus*; PDB, Protein Data Bank; *Pm*TyvE, CDP–tyvelose 2-epimerase from *Persephonella marina*; *St*TyvE, CDP–tyvelose 2-epimerase from *Salmonella typhi*; *Ta*TyvE, CDP–tyvelose 2-epimerase from *Thermodesulfatator atlanticus*; TyvE, CDP–tyvelose 2-epimerase; *Yp*TyvE, CDP–tyvelose 2-epimerase from *Yersinia pseudotuberculosis*.

The three-dimensional structure of *Ta*TyvE was obtained from a protein crystal grown in the presence of NAD^+^ and CDP. The structure was determined by molecular replacement based on the reported structure of the TyvE from *S. typhi* (*St*TyvE) in complex with NAD^+^ and CDP (PDB ID: 1ORR) (23). Data collection and refinement statistics are shown in Table S1, and the crystal structure was deposited in the PDB (PDB ID: 9RL0). *Ta*TyvE crystallized with eight polypeptide chains in the asymmetric unit. The crystalline packing arrangement revealed two spatially separated tetrameric assemblies of protein protomers. All eight protomers (A–H) were highly similar to each other, indicated by an RMSD for the α-carbon traces of 0.253 Å (2253 atoms) to 0.980 Å (2124 atoms) with respect to protomer A (Fig. S2*A*). Each tetramer featured D2 symmetry and was best described as a dimer of dimers. Gel-filtration profiles across varying enzyme concentrations showed a dominant peak with only a minor higher–molecular-weight component, consistent with one stable oligomeric form (Fig. S3). These findings agree with native mass spectrometry data (Fig. S4), confirming a tetrameric assembly for *Ta*TyvE. Enzyme dimers A/B and C/D follow the structural principle of a four-helix bundle that is well known for the assembly of SDR enzymes into their functional oligomers (Figs. 2*B* and 3*A*) (2, 23, 25, 43, 45). The α-helices α5 (Pro92–Tyr113) and α6 (Pro163–Tyr182) from each protomer contribute to the dimer interface and are arranged symmetrically to interact in a parallel fashion with one another (Fig. 2, *A* and *B*).

*Ta*TyvE (340 residues) is 65% identical in sequence to *St*TyvE (339 residues) (23). Comparing the enzyme protomers bound with NAD^+^ and CDP, the overall structure is found to be highly similar for both enzymes (RMSD = 0.479 Å; 1982 atoms; Fig. S2*B*). The crystalline assembly into a dimer of dimers is almost identical for *Ta*TyvE and *St*TyvE (23), as shown in Figure S2, *C* and *D*. *St*TyvE was furthermore reported to be a tetramer in solution based on ultracentrifugation experiments (23).

The protomers of each dimer A/B and C/D are placed side by side in a back-to-back orientation. In contrast to the well-formed interactions within each dimer, there are additional interactions between the two dimers *via* protomers A/C and B/D. The polypeptide region Asn32 to Tyr39 and α-helix α3 (Gly56–Lys66) are the main contributors to the interprotomer interactions along the A/C and B/D pairs (Figs. 2, *B* and *C* & 3*A*). Both regions originate from the NAD^+^-binding Rossmann fold domain of the protomer. Analysis of the structure data with PDBePISA (46) revealed a buried interfacial surface area for A/B and C/D calculated as 1384.1 Å^2^ and 1389.7 Å^2^, respectively (∼9% of total protomer surface area). By way of comparison, the buried surface area at the interface of the A/C and B/D pairs was much lower, with values of 205 Å^2^ and 219 Å^2^, respectively. Interestingly, occupancy of the NAD^+^ and CDP ligands was not uniform across the enzyme protomers of the tetrameric assembly. In the A/B dimer, protomer B contained NAD^+^ but no CDP, whereas protomer A contained both NAD^+^ and CDP. In the C/D dimer, each protomer contained NAD^+^, but CDP was absent in both. The same distribution of NAD^+^ and CDP ligands was observed for protomers E to H in the second tetramer: protomer E contains both NAD^+^ and CDP, whereas protomers F to H lack the latter.

Discussion of the tertiary structure of *Ta*TyvE refers to protomer A that has both NAD^+^ and CDP bound to the enzyme. The overall molecular architecture of the protomer involves two distinct structural domains (Figs. 2*C* and 3*A*): The N-terminal Rossmann fold domain for the binding of NAD^+^ and the C-terminal domain, which has a simpler fold and is rich in loop elements, for the binding of the CDP–(sugar) (2, 3, 4). The active site is located in the cleft between the two domains. The triad of residues of the SDR catalytic signature motif for oxidation and reduction is contributed by the Rossmann fold domain (Thr124, Tyr164, and Lys168), as shown in Figures 2, *C* and *D* and 3*A* (2, 4).

The Rossmann fold domain (Figs. 2*C* and 3, *A* and *B*) consists of a single seven-stranded parallel β-sheet (β1–5, β8, and β13) connected by seven α-helices (α1–6, α8) and various loop regions.

The residues for NAD^+^ binding (Fig. S5*A*) are identical in both enzymes, with the exception that Ser34 adjacent to Arg35 of *St*TyvE (23) is substituted by Tyr34 in *Ta*TyvE. The β-hairpin loop (Gly132–Asn150; Fig. 3*A*) was found to be structurally conserved in TyvE-like enzymes (47).

The C-terminal domain (Figs. 2*C* and 3, *A* and *B*) consists of a mixed β-sheet formed by three strands (β11–12 and β14, β9), a parallel β-sheet formed by two strands (β10, β15), and four major α-helices (α7, α10–12).

The binding pocket for CDP is shown in Figure S5*B*. The surrounding residues are identical in *Ta*TyvE and *St*TyvE (23). Notably, Lys126 is flexible in all enzyme complexes, with various rotamers seen in protomers A to D and E to H (Fig. S8, *A* and *E*).

Further comparison of *Ta*TyvE to characterized TyvEs (6, 23, 47) by both structural and sequence alignments (Fig. 3) reveals a high level of conservation for residues involved in binding NAD^+^ and the CDP–(sugar). Moreover, active-site residues within the sugar-binding pocket of *Ta*TyvE (Fig. 3*C*) are superimposable across all enzymes analyzed (Fig. 3*D*).

### Induced-fit conformational change because of the binding of CDP

**Figure 4:**
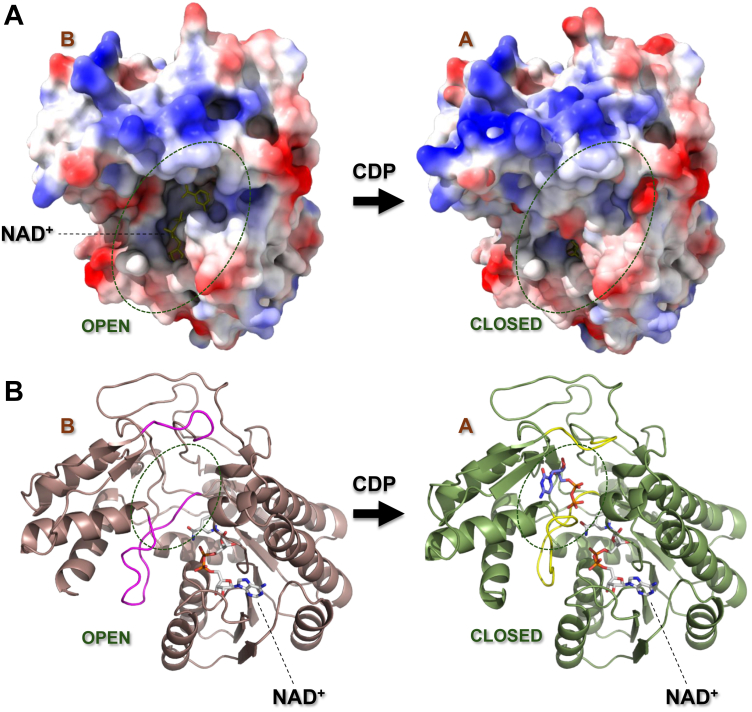
**Induced-fit conformational change in *Ta*TyvE by CDP binding.***A,* electrostatic surface representation of protomer B complexed with NAD^+^ (*open loop*) and protomer A bound to CDP in the ternary complex (*closed loop*). *B, cartoon representation* of both protomers A and B (labeled). Loop regions Pro297 to Asp302 (*top*) and Ser192 to Trp207 (*bottom*) are highlighted in *pin*k in the open loop conformation (protomer B). Protomer A in complex with NAD^+^*(gray*) and CDP (*blue*), with closed loops colored in *yellow*. *Ta*TyvE, CDP–tyvelose 2-epimerase from *Thermodesulfatator atlanticus*.

*Ta*TyvE protomers (B–D, F–H) lacking bound CDP consistently exhibit low electron density for the polypeptide chain lying between Gly197 and Trp207 (Fig. S6, *A* and *H*). Interpretation of the electron density in terms of a defined loop structure was possible only for protomer B, as shown in Figure S6*B*. The Gly197–Trp207 region of the sequence belongs to a larger loop (Ser192–Trp207) that is located in the cleft between the N- and C-terminal domains (Fig. 3*A*) (48, 49, 50, 51). In the NAD^+^–CDP complex, by contrast, the Gly197–Trp207 region is well ordered (Fig. 3*A*). Electrostatic surface and cartoon representations of protomers B (open-loop conformation) and A (closed-loop conformation) are shown in Figure 4. In protomer A, the Gly197–Trp207 loop folds inward, closing off and constraining the CDP–(sugar) binding pocket from below as it approaches helix α7 (Fig. 4). At the top of the CDP–(sugar) binding pocket, a polypeptide chain (Pro297–Asp302; Figs. 4; S6*J*), which is part of a larger, N- and C-terminal domain–connecting loop (Asp288–Asp309) is found. The “top loop” is disordered in the majority of protomers in the absence of CDP but not universally so: protomer F adopts an ordered conformation (Fig. S6*J*), whereas protomers B–D and G–H crystallized disordered. Note that crystal packing or bound artifacts may influence local conformation. However, in the CDP-bound structure (Figs. 4, S6*J*), this loop moves down to close off the pocket from the top. The “bottom loop” (Gly197–Trp207), by contrast, requires bound CDP to become structurally ordered (Figs. 3*A*, 4*B*), implying an induced-fit conformational change.

At the single-residue level, the loop rearrangements caused by CDP binding result in large positional displacements for Tyr203, Asp204, and Gln205 that coconfine the CDP–(sugar) binding pocket (Figs. 3*C*, S7). Tyr203 and Asp204 close over the cytosine moiety of CDP, whereas the Gln205 side chain appears positioned to interact with the sugar moiety (Fig. S7). Smaller changes are seen for Gly206 (Fig. S8*D*). The main chains of Ser192 and Ser193 are displaced by ∼2.2 Å (Fig. S8*F*).

A comparison of *Ta*TyvE and *St*TyvE active sites reveals that the homologous loop region in the ternary complex is superimposable (23) (Fig. S7*D*). However, the NAD^+^ complex structure without CDP is not known for *St*TyvE (23). Note that local conformational variability is suggested in the closed state of *St*TyvE, as different enzyme protomers adopt distinct CDP conformers that vary in the orientation of the pyrophosphate moiety, the rotamer state of Gln205, and the conformation of the Pro297–Asp302 loop, including Arg299 (Fig. S7, *C* and *D*).

Contrasting the flexibility of the C-terminal domain, the N-terminal domain of *Ta*TyvE is relatively more rigid and structurally unaffected by CDP binding (Fig. S8). Within the N-terminal domain, the spatial arrangement of the catalytic triad (Tyr164, Thr124, and Lys168) appears to be highly preorganized: it is identical in enzyme complex structures with NAD^+^ (protomers B–D and F–H) and NAD^+^/CDP (Fig. S8, *A* and *B*).

### Substrate binding analyzed by molecular docking and MD simulations

Molecular docking was performed with the whole molecule of CDP–Glc or CDP–Man used as a ligand of enzyme–NAD^+^ (protomer A with CDP removed). Docking poses for each receptor–ligand complex were evaluated on the criteria that residue displacements stay within the experimental limits of positional variation seen in protomers A–H and that the pyrophosphate-linked sugar is placed plausibly for catalytic oxidation by NAD^+^.

The generated ternary complexes were modeled into the tetrameric assembly of *Ta*TyvE and served as the starting configurations for classical MD simulations. Protein residues and sugar moieties were described using the Amber19SB and GLYCAM06j-1 force fields, respectively. Substrate geometries were optimized at the BP86/def2-SVP level, and RESP charges were derived at the HF/6-31G∗ level. Protonation states at pH 7.5 were assigned with H++. After solvation and neutralization, complexes were minimized, heated to 333 K, equilibrated, and subjected to 1.5 μs MD simulations. Trajectories were analyzed with cpptraj to obtain heavy-atom distances, dihedral angle distributions, ring puckering, and free energy profiles (see MD simulations for method details and Figs. S9–S11 for results).

#### Distance-based evaluation of catalytically plausible poses

To assess whether catalytically plausible poses were sampled, heavy-atom distances ≤3.5 Å were evaluated according to the requirements of the canonical SDR mechanism of alcohol oxidation (2, 3). Configurations were considered catalytically plausible when the phenolic oxygen of Tyr164 (the catalytic base) and the Thr124 side-chain oxygen were within hydrogen-bonding distance of the reactive sugar C2 oxygen. In addition, the Lys168 NZ atom had to interact with the ribose hydroxyl oxygen atoms of NAD^+^. The distance between the nicotinamide C4 atom of NAD^+^ and the sugar C2 had to be compatible with hydride transfer (≤3.5 Å). Analysis of the full MD ensemble showed that 1.6% and 4.18% of frames for CDP–Glc and CDP–Man, respectively, met the defined criteria. For comparison, average heavy-atom distances across the full MD ensemble (24 × 10^3^ frames) are shown in Figure S9.

Using a 3.5 Å cutoff, we analyzed residues capable of interacting with the C3 and C4 oxygen atoms in both binding poses. For Asn125 and Gln205, distances were measured from the side-chain amide carbon atoms (Asn125 CG; Gln205 CD) to the sugar hydroxyl oxygen atoms, as the relative orientation of the amide oxygen and nitrogen could not be predefined. A 4.0 Å cutoff was applied for these calculations.

For CDP–Glc, proximity of Asn125 (CG) to the C3 oxygen was observed in 0.18% of catalytically plausible poses, whereas no contacts with the C4 oxygen were detected. In 0.08% of frames, Asn125 approached either the C3 or C4 oxygen in conformations that did not satisfy the criteria of the canonical SDR alcohol oxidation mechanism.

For CDP–Man, Gln205 (CD) approached the C4 oxygen in 0.06% of frames and the C3 oxygen in 0.33% of frames. The Val83 backbone carbonyl oxygen contacted the C3 oxygen in 3.13% of frames and the C4 oxygen in 3.86% of frames. The Met85 backbone carbonyl oxygen approached the C4 oxygen in 0.08% of frames. Notably, the average heavy-atom distances sampled throughout the simulations (Fig. S9, *A*–*C*) indicate that more than 94% of conformations did not meet the defined criteria (distance >3.5 Å) and therefore represent unproductive poses. The C2–H2–C4(NAD^+^) angle for hydride transfer calculated from the 3.5 Å cutoff MD ensemble is typically ∼130 to 140° on average, with a maximum of ∼162°.

**Figure 5:**
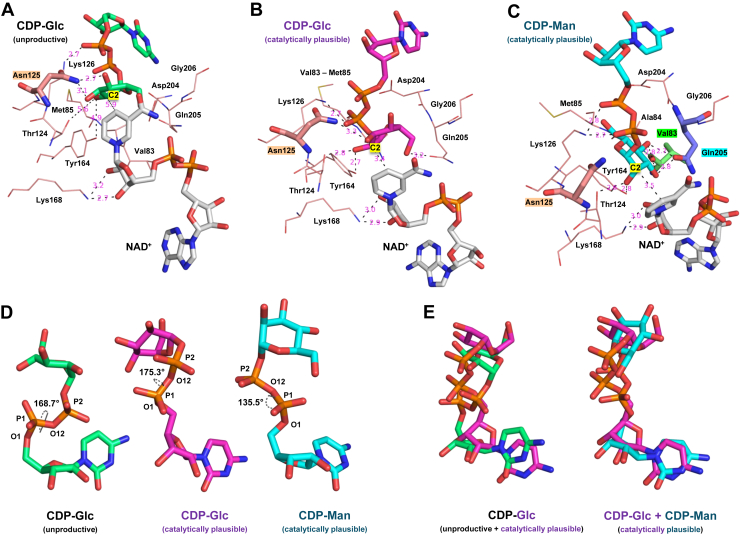
**MD-derived *Ta*TyvE complexes with CDP–Glc and CDP–Man and associated pyrophosphate torsion.***Ta*TyvE in complex with (*A*) CDP–Glc (unproductive), (*B*) CDP–Glc (catalytically plausible), or (*C*) CDP–Man (catalytically plausible) is shown. NAD^+^ is depicted in *gray*, and heavy-atom distances are depicted in *magenta*. *D,* extracted substrate poses corresponding to *A*–*C*, as well as their overlay (*E*), are displayed. The dihedral angle between O1–P1–O12–P2 of the pyrophosphate backbone is indicated. MD, molecular dynamics; *Ta*TyvE, CDP–tyvelose 2-epimerase from *Thermodesulfatator atlanticus*.

Figure 5 shows catalytically plausible and unproductive binding modes of CDP–Glc and CDP–Man in the *Ta*TyvE active site, along with the corresponding pyrophosphate torsions. Figure 5*A* depicts an unproductive CDP–Glc complex. Although Asn125 is within hydrogen-bonding distance to the C3–OH or C4–OH (≤3.1 Å), oxidation at the C2 is not supported, as both the Tyr164 phenolic hydroxyl and hydride transfer distances exceed 3.5 Å. Figure 5*B* shows a catalytically plausible pose of CDP–Glc. Heavy-atom distances within the catalytic triad (Tyr164, Thr124, and Lys168) are ≤3.0 Å, and the hydride donor–acceptor distance is 3.4 Å. The C2–H2–C4(NAD^+^) angle is 138.3°. In addition, the Asn125 side chain interacts with the C3–OH of glucose but does not contact the C4–OH. A catalytically plausible pose of CDP–Man is illustrated in Figure 5*C*. Heavy-atom distances within the catalytic triad are ≤3.0 Å. The hydride donor–acceptor distance measures 3.5 Å, with a C2–H2–C4(NAD^+^) angle of 130.7°. Moreover, either the Gln205 side chain or the Val83 backbone carbonyl contacts a single mannose hydroxyl at C3 or C4 (≤2.8 Å). Note: As the C6–OH is not required for catalytic turnover (17), Figure 5 shows only pose-relevant contacts. In CDP–Glc, C6–OH interacts with the Gln205 side chain (Figs. 5*B* & S9*C*), whereas in CDP–Man, it contacts the backbone of Val83 or Met85 (Fig. S9*C*).

#### Pyrophosphate torsion and low-energy states

To assess the orientation of the pyrophosphate moiety, we identified the most frequently sampled O1–P1–O12–P2 dihedral angle (Fig. 5*D*) as a stable reference parameter across the full MD ensemble. The pyrophosphate dihedral associated with CDP–Glc accumulates around ∼170° (±5°), whereas that associated with CDP–Man accumulates around ∼130° (±5°) (Fig. S11*E*). In Figure 5, the pyrophosphate dihedral angle is 168.7° for unproductive CDP–Glc binding (Fig. 5, *A* and *D*), 175.3° for catalytically plausible CDP–Glc (Fig. 5, *B* and *D*), and 135.5° for catalytically plausible CDP–Man (Fig. 5, *C* and *D*). An overlay of the substrate poses is presented in Figure 5*E*.

The Pα and Pβ of the pyrophosphate moiety are tightly coordinated by the Lys126 side chain, which is equidistant to the P1 and P2 phosphorus atoms in both configurations (Fig. S9*B*). Pα, furthermore, interacts with Arg237 and Arg299. Note that interaction refers to dynamic contacts observed in MD simulations, in which both residues transiently engage the α-phosphate through electrostatic interactions and associated hydrogen bonds, which are not apparent in the static crystal structure (Fig. S5*B*).

Catalytically plausible poses were further analyzed by deriving two-dimensional free-energy landscapes from the MD simulations. The pyrophosphate torsional angle and the heavy-atom distance between the Tyr164 phenolic oxygen and the sugar C2 oxygen were used as collective variables (Fig. S11, *A* and *B*). For CDP–Glc, the lowest-energy regions are centered at larger Tyr164–O–C2–O distances (∼4.8 and ∼7.3 Å). These basins coincide with the most populated pyrophosphate dihedral angles (Fig. S11*E*). Conformations within hydrogen-bonding distance are therefore less frequently sampled. These findings are thus consistent with previous calculations showing that only 1.6% of the MD ensemble satisfies the criteria of the SDR mechanism of alcohol oxidation. In contrast, CDP–Man occupies low-energy regions centered at ∼3 and ∼5 Å. Both basins fall within the most frequently sampled range of pyrophosphate dihedral angles (Fig. S11*E*). This aligns with 4.18% of CDP–Man poses meeting the criteria for oxidation, exceeding the fraction observed for CDP–Glc.

#### Puckering analysis

The extent of sampling observed in the two-dimensional energy profiles reflects variation along the selected collective variables and does not capture additional conformational degrees of freedom outside this coordinate space. Hence, we extracted hexopyranosyl moieties of CDP–Man and CDP–Glc from MD simulations of the enzyme-bound complexes and analyzed the puckering of the sugar moiety. Puckering analysis along the classical MD simulations reveals that the CDP–Man hexopyranosyl moiety shows a dominant ^4^*C*_1_ chair conformation; however, there are additional populations toward ^1^H_0_, ^1^E, and ^1^H_2_, as well as a minor cluster near the ^0^S_2_ region (Fig. S10*A*). For CDP–Glc, the conformational ensemble is more tightly confined to the ^4^*C*_1_ basin (Fig. S10*B*).

#### Evaluation of docking-initiated MD simulations

To assess the validity of the MD simulations initiated from the docking poses, additional simulations were conducted for both substrates (CDP–Glc, CDP–Man); however, they employed the CDP moiety accommodations observed in the crystal structure from *St*TyvE. Although mesophilic *St*TyvE has not been experimentally shown to catalyze epimerization reactions with these substrates, the pronounced differences in electrostatic surface charge distribution (Fig. S12) may already suggest structural dynamics distinct from those of thermophilic *Ta*TyvE.

Our simulations were designed to examine how adopting the *St*TyvE-derived CDP poses influence substrate sampling when introduced into *Ta*TyvE under otherwise identical MD protocols. The free-energy landscapes (Fig. S11, *A*–*D*) show that both CDP–Glc and CDP–Man sample multiple substrate poses in *Ta*TyvE, irrespective of whether the initial structures were derived from docking solutions (Fig. S11, *A* and *B*) or from the *St*TyvE-based CDP orientations (Fig. S11, *A* and *C*). Notably, simulations initiated from the *St*TyvE-derived CDP even explore a broader conformational space, particularly for CDP–Man (Fig. S11*D*). Low-energy regions for the Tyr164–O–C2-O distance span approximately 3 to 8 Å. For CDP–Glc, the minima are centered at ∼5.5 and ∼7 Å (Fig. S11*C*). These broad low-energy basins coincide with wide distributions of the corresponding pyrophosphate dihedral angles (Fig. S11*F*). CDP–Glc shows a minor accumulation near ∼130°, whereas CDP–Man populates two distinct dihedrals around ∼60° and ∼170°. In contrast, the docking-derived simulations (Fig. S11*E*) converge to comparatively sharply defined and substrate-specific dihedrals with minimal overlap. Hence, this comparison indicates that the docking-derived starting structures do not impose artificial constraints but instead allow resolution of the intrinsic conformational preferences of the bound substrates.

### Enzyme specificity probed with mutagenesis and substrate analogs

Site-directed mutagenesis is limited in its ability to probe the role of main-chain interactions. Therefore, we additionally used substrate analogs for the analysis of enzyme specificity. Individual synthetic replacements of sugar hydroxyl groups with hydrogen or fluorine have proven useful in the characterization of hydrogen bonding interactions with the substrate in carbohydrate-active enzymes (52, 53, 54, 55, 56).

#### Mutagenesis

Considering their proposed roles in stabilizing both substrate conformations *via* side-chain interactions, Asn125 and Gln205 were of principal interest for mutagenesis. Results are shown in Table 1. The reference activity of wildtype *Ta*TyvE for epimerization of CDP–Glc was 23.1 mU mg^−1^. The N125A variant was inactive, whereas the Q205A variant showed only 12-fold decreased activity compared with the wildtype enzyme.

**Table 1:** Specific activities of *Ta*TyvE variants with CDP–Glc used as substrate

*Ta*TyvE *variant*	Specific activity (mU mg^−1^)
Wildtype	23.1 (±5%)
V83A	16.0 (±5%)
V83S	17.9 (±5%)
M85A	15.5 (±7%)
N125A	ND
K126A	ND
Q205A	1.9 (±7%)

ND, not detectable.

Reactions were conducted in MOPS buffer at 60 °C (pH 7.5). Experiments were performed in triplicate (*N* = 3).

Lys126 was also substituted because of its proposed interaction with the pyrophosphate moiety in both rotational endpoints (Figs. 5 & S9*B*) and conservation in TyvEs (47, 57). The K126A did not show detectable activity. Backbone interactions (Figs. 5*C* & S9*C*) of the *manno*-substrate, as suggested by MD simulations, were probed by alanine substitutions at Val83 and Met85. Both variants, as well as V83S, retained activity comparable to the wildtype enzyme.

#### Substrate analogs

The approach by substrate analogs targeted the C4 and C6 of the substrate. Since Gln205 interacts with the *manno*-C4- or *gluco*-C6-hydroxyl groups (Fig. 5, *B* and *C* & S9*C*), we reasoned that reducing substrate positioning flexibility in the closed active site—as indicated by MD simulations—might affect the reaction rate. Extending our earlier research that used synthetic CDP–6-deoxy-Glc to probe the reactivity with *Ta*TyvE (17), we obtained here CDP–d-lyxose (CDP–Lyx) as a C5 analog of CDP–Man that has the C6 hydroxymethyl group removed. Two-step enzymatic synthesis failed despite extensive efforts (Supporting Information), and so we established chemical coupling of Lyx and CDP using 2-chloro-1,3-dimethylimidazolinium chloride as the promoting agent (∼20% conversion). CDP–Lyx (∼3.6 mg) was recovered in high purity (UV_271 nm_ ∼99%). Capillary zone electrophoresis (CE) traces and NMR data of the isolated CDP–sugars are shown in the supporting information (Figs. S13–S17).

We further examined the role of the sugar C4–OH: hydrogen bonding to the native CDP–Par/CDP–Tyv pair is limited to the reactive sugar C2–OH and a single additional C4-hydroxyl group (Fig. 1*A*) (17, 23)*,* suggesting that the C4–OH is a key determinant for substrate positioning. Syntheses of CDP–4-deoxy-α-d-glucose (CDP–4-deoxy-Glc) and CDP–4-deoxy-4-fluoro-α-d-glucose (CDP–4-fluoro-Glc) were performed as described in the Experimental procedures section and the supporting information. The compounds (∼1 mg) were isolated in sufficient purity for reaction with the enzyme. CDP–galactose (CDP–Gal) is the C4 epimer of CDP–Glc and was prepared (3.5 mg) from Gal. Results are shown in Table 2.

**Table 2:** Specific activities of the *Ta*TyvE wildtype enzyme with substrate analogs

Substrate analogs	Specific activity (mU mg^−1^)
CDP–Glc	23.1 (±5%)
CDP–4-deoxy-Glc	ND
CDP–4-fluoro-Glc	9.8 ∙ 10^−3^
CDP–Gal	ND
CDP–Lyx	5 ∙ 10^2^ (±6%)

ND, not detectable.

Reactions were conducted in MOPS buffer at 60 °C (pH 7.5). Where applicable, experiments were performed in triplicate (*N* = 3).

*Ta*TyvE was inactive with CDP–4-deoxy-Glc and CDP–Gal despite the use of 1 mM enzyme (40 mg ml^−1^) in the reaction. A small amount of activity was recovered when CDP–4-fluoro-Glc was used (Table 2). The CDP–4-fluoro-Glc preparation contained CTP (21.7 mass%). We evaluated with CDP–Glc that the same amount of CTP would give a 3.4-fold decrease in activity to 6.8 mU mg^−1^, suggesting a ∼10^3^-fold lowering of *Ta*TyvE activity as a result of substitution of the C4-hydroxyl group of glucose with fluorine (Fig. S18). Interestingly, epimerization of CDP–Lyx to CDP–Xyl proceeded at 14-fold elevated specific activity (Table 2) compared with the epimerization of CDP–Man to CDP–Glc (36 mU mg^−1^; ±5%; (17)).

## Discussion

### Structural preorganization linked to the catalysis of *Ta*TyvE

Evidence was presented that structural preorganization toward the *Ta*TyvE Michaelis complex with CDP–Glc–CDP–Man involves induced fit by binding of the CDP portion of the substrate. The protein conformational changes by CDP binding are localized within the C-terminal domain of the *Ta*TyvE protomer. Overall, they result in a structural ordering and compaction of the cleft region between the two structural domains (Fig. 4). The Gly197–Trp207 loop is the main structural element of induced fit: it undergoes a transition from a flexible to a well-ordered structure and moves into the interdomain cleft to confine the size and shape of the substrate binding pocket. Remarkably, it does so without developing noticeably strong, direct interactions with either the CDP or the hexopyranosyl moiety of the substrate. The apparent paucity of enzyme–substrate noncovalent bonds might be explained by the torsional freedom that is required to accommodate multiple poses of the CDP's pyrophosphate (Fig. 5). Comparison of *Ta*TyvE to sugar nucleotide C4 epimerases (GalE (26), GlcNAcE (30, 31), and GlcAE (33)) reveals conserved features with regard to the interplay of conformational flexibility and structural preorganization in substrate binding and catalysis. All enzymes show substantial loop rearrangements at the interdomain cleft by nucleoside diphosphate binding during formation of the Michaelis complex. However, only minor and locally restricted conformational changes are required for the torsional motions of the ketointermediate during the rotation step of the epimerase catalytic cycle (27, 30, 33, 44, 58, 59).

Among TyvE-like epimerases (Fig. 3*B*) (17, 47) and (putative) CDP–glucose 4,6-dehydratases (17), VAM (Val83–Ala84–Met85) and TNK (Thr124–Asn125–Lys126) segments are highly conserved. The VAM segment, together with Gln205, contributes to stabilization of CDP–Man (Figs. 5*C* & S9). Notably, the residue equivalent to position 83 in *Ta*TyvE has been implicated in substrate positioning in related epimerases, involving GlcAE (Fig. S1; Pro85), GalE (Lys84). and UDP-xylose 4-epimerase (Ala156) (60).

Mutation of the TNK segment (N125A, K126A) resulted in complete loss of activity with CDP–Glc. While Thr124 forms part of the catalytic triad and Lys126 interacts with the pyrophosphate moiety in both substrate poses (Fig. S9*B*), Asn125 likely contributes critically to glucose positioning.

### Glucose–mannose substrate specificity of *Ta*TyvE

#### Reactivity with CDP–Glc

Analysis of the full MD ensemble (Fig. S9*C*) shows that the average heavy-atom distances between Asn125 (CG) and the C4-O (4.6 ± 0.9 Å) or C3-O (5.3 ± 1.3 Å) of glucose are comparable. Within the 3.5 Å cutoff ensemble, proximity of Asn125 to either hydroxyl group alone does not necessarily correspond to catalytically plausible poses and is also observed in unproductive CDP–Glc conformations (Fig. 5*A*). A small fraction of the analyzed MD frames (0.18%) satisfies the criteria for alcohol oxidation while exhibiting an Asn125–C3–O interaction (Fig. 5*B*). Our previous study (17) demonstrated that CDP–3-deoxy-Glc is unreactive with *Ta*TyvE, supporting the functional importance of the C3–OH group for catalysis. These findings indicate a finely tuned network of hydrogen-bonding interactions involved in catalytically plausible binding, raising the question of how modifications at C4 influence this balance.

We thus hypothesize that the reduced or abolished reactivity of C4-deoxygenated and C4-fluorinated CDP–Glc analogs arises from disruption of interactions that modulate access to catalytically plausible conformations. In this context, removal of the C4-hydroxyl group would increase the probability of sampling unproductive poses, consistent with the complete loss of activity observed for the N125A variant. The C4-fluoro analog may still populate ^4^*C*_1_ chair conformations in the *gluco*-configuration, in line with reports that fluorination of the pyranosyl moiety does not significantly distort the chair conformation (56, 61). Moreover, the glucosyl moiety more readily adopts minimally distorted ^4^*C*_1_ chair conformations than mannose (Fig. S10). Consequently, unfavorable electrostatic interactions with main-chain carbonyls of the VAM segment in the *manno*-configuration are likely to largely decrease its reactivity. In contrast, the C4 inverted CDP–Gal imposes steric constraints that restrict access to catalytically plausible conformations, rather than perturbing electrostatic interactions.

Notably, in *St*TyvE, the Asn125 side chain has also been proposed to interact with the C4-hydroxyl of CDP–Par (23). The TyvE from *Y. pseudotuberculosis* (*Yp*TyvE), which derives from a mesophilic organism like *St*TyvE and shares higher sequence identity with *St*TyvE (71.9%) than *Ta*TyvE (66.1%), was shown to be inactive with CDP–glucose (6). However, in *Yp*TyvE, and similarly to *Ta*TyvE, substitution of the C4-hydroxyl group with fluorine in CDP–Par also resulted in a substantial decrease (∼31-fold) in enzymatic activity relative to CDP–Par (6). While CDP–Par lacks the C3 and C6 hydroxyl groups, these observations suggest that C4 interactions contribute more generally to organizing substrate conformations within the active site.

#### Reactivity with CDP–Man

MD-derived interactions in the ternary complex (Fig. 5*C*) suggest a conserved interaction also observed in *St*TyvE. In both enzymes, Gln205 contacts the C4–OH of CDP–Man (*Ta*TyvE) or modeled CDP–Tyv (*St*TyvE) (23). However, the moderate activity loss in the Q205A variant indicates that catalytically plausible positioning of CDP–Man is not governed by a single side-chain interaction. MD simulations (Figs.S9*C*, S10*A*, S11*B*) suggest that the backbone carbonyl oxygens of the VAM segment interact with CDP–Man, particularly Val83 (Fig. 5*C*). This is supported by minor activity changes in the V83A, V83S, and M85A variants. Accordingly, greater conformational flexibility of CDP–Man is implied, consistent with the increased distortion of its ^4^*C*_1_ chair conformations relative to glucose (Fig. S10*A*). This enhanced flexibility allows CDP–Man to fulfill the catalytic distance criteria more frequently than CDP–Glc (4.18% *versus* 1.6%) and to adopt more favorable positions along the Tyr164–O–C2–O coordinate (Fig. S11*A*).

#### Insights into CDP–Man reactivity from CDP–Lyx

Attempts to synthesize CDP–Par and CDP–Tyv *via* chemoenzymatic methods were unsuccessful despite extensive efforts (17), precluding direct comparison of corresponding activity data (6). However, CDP–Lyx, given its simpler structure relative to CDP–Man and closer resemblance to CDP–Tyv, might be regarded as a structural analog to probe electrostatic or steric constraints of the *Ta*TyvE active site. Interestingly, the hydroxymethyl substituent at the C5 is not needed for catalytic positioning, and the rate coefficient of CDP–Lyx (*k* = 19.6 min^−1^) closely matches the reported *k*_cat_ value (∼22 min^−1^) for CDP–Par/Tyv with *Yp*TyvE (6). These observations point to a similar active site volume in both enzymes under conditions involving less bulky substrates. Although active site residues are identical (Fig. 3, *B* and *D*), the inability of *Yp*TyvE to utilize CDP–Glc (6) suggests that differences arise from how the substrate is accommodated within the active site. In contrast to the parent C4-fluorinated analog, CDP–Lyx maintains favorable electrostatic interactions with *Ta*TyvE in both binding poses. Moreover, the absence of the hydroxymethyl group in CDP–Lyx diminishes its interactions with the VAM backbone carbonyls in the *manno*-configuration (Fig. S9*C*). This reduces conformational flexibility and facilitates more efficient turnover compared with CDP–Man. Together, these results suggest that CDP–Man samples a disproportionate number of unproductive poses relative to catalytically plausible ones, which may account for its lower reactivity relative to CDP–Lyx.

#### Crystallographic observations: The Gln205 side chain

A tentative model for the differential substrate specificity of thermophilic and mesophilic TyvEs is that the flexible Gly197–Trp207 loop governs the active site volume and, consequently, the extent of electrostatic interactions with the VAM and TNK segments. In the crystal structure of the *Ta*TyvE ternary complex, Gln205 adopts a single, well-defined side-chain configuration within this loop. In contrast, in *St*TyvE, the Gln205 side chain also samples orientations directed away from the sugar moiety (Fig. S7, *C* and *D*). Although this observation does not establish global differences in active site flexibility, it may indicate enhanced local conformational fluctuations within the Gly197–Trp207 loop of the mesophilic enzyme.

#### Electrostatic features: Surface charge distribution

Distinct differences in surface charge distribution between *St*TyvE and *Ta*TyvE are evident (Fig. S12). Surface charge distribution has been shown to affect local flexibility, including the active site, by altering electrostatic interactions, as well as hydration behavior and solvation dynamics in differently temperature-adapted subtilisin-like serine proteases (62). Furthermore, a differing (“softer”) surface potential has been associated with increased reactant configurational space for enzymes adapted to lower temperatures (63). Hence, different orientations observed for Gln205 in *St*TyvE may reflect fluctuations in the surrounding electrostatic environment that modulate local loop dynamics, rather than intrinsic residue-specific effects.

#### MD observations: conformational sampling

MD simulations initiated from the *St*TyvE-derived CDP orientations sample a broader ensemble of low-free-energy conformations (Fig. S11, *C* and *D*). This suggests that even subtle variations in the initial substrate pose can increase the conformational space explored within the active site. However, such broadened sampling reduces the relative population of catalytically plausible states (Fig. S11, *C* and *D*). This parallels the pattern of CDP–Man/CDP–Lyx discussed above, where unproductive states likely constitute a larger fraction of the sampled ensemble. Hence, structural variability within flexible regions, such as the Gly197–Trp207 loop, may significantly modulate the accessible conformational space, with consequences for substrate positioning and specificity.

At this point, it is important to recall that more than 94% of conformations sampled in the full MD ensemble correspond to unproductive poses. The remaining ∼6% fulfill the catalytic criteria, involving interactions with the catalytic triad alone or in combination with residues, such as Gln205, Asn125, or Val83. The active site of thermophilic *Ta*TyvE may be more rigid, thereby restricting the accessible conformational space. This allows a small but defined fraction of poses to sustain catalysis of the challenging CDP–Glc/CDP–Man epimerization. In mesophilic homologs, by contrast, increased flexibility may favor unproductive conformations, thereby reducing the relative population of catalytically plausible states.

In summary, the *Ta*TyvE crystal structure reveals an induced-fit conformational change that is required for substrate accommodation. Functional analyses, supported by mutagenesis, MD simulations, and mechanistic probes, implicate VAM and TNK segments in mediating the epimerization between CDP–Glc and CDP–Man. Differences in substrate specificity arise from differential conformational sampling in a confined active site. These insights highlight the critical balance between structural preorganization and conformational flexibility as a key determinant in *Ta*TyvE catalysis.

## Experimental procedures

### Materials

Nucleotides, d-glucose, d-lyxose, and d-galactose were from Carbosynth. 4-Deoxy-d-glucose and 4-deoxy-4-fluoro-d-glucose were from Omicron Biochemicals, Inc. Deuterium oxide (99.96% ^2^*H*) was from Euriso-Top. All other chemicals and reagents were of the highest available purity. *Escherichia coli* BL21(DE3) competent cells were prepared in-house. Q5 High-Fidelity DNA polymerase and DpnI were from New England Biolabs. Oligonucleotide primers were from Sigma–Aldrich. A GeneJET Plasmid Miniprep Kit (Thermo Scientific) was used for plasmid DNA isolation.

### Enzyme production

#### Production of *Ta*TyvE variants

*Ta*TyvE variants were prepared using a modified QuikChange protocol using wildtype plasmid as the template. PCRs were carried out in a reaction volume of 50 μl using 20 ng of plasmid DNA as a template and 0.2 μM of forward or reverse primer. Q5 DNA polymerase was used for DNA amplification. PCR products were transformed into electrocompetent *E. coli* BL21 (DE3). Plasmid DNA was extracted and sequenced with T7prom–T7term primers provided by LGC Genomics to confirm mutations. Enzymes were expressed in *E. coli* BL21 (DE3) harboring a pET21a expression vector with the respective gene. A C-terminal 6xHis-tag was used for enzyme purification. Full details on PCR protocols, enzyme expression, and purification are given elsewhere (17, 24).

#### Production of enzymes for substrate synthesis

Galactokinase from *Streptococcus pneumoniae* (GalKSpe4; EC 2.7.1.6; (64)) was produced in *E. coli* BL21 (DE3) LEMO21 cells carrying a pET28a_GalKSpe4 vector. Galactokinase from *E. coli* (GalK; EC 2.7.1.6; (65)) was expressed in *E. coli* BL21 (DE3) harboring a pET-STREP3_GalK vector. Inorganic pyrophosphatase from *E. coli* (iPPase; EC 3.6.1.1; (66)) was expressed in *E. coli* BL21 (DE3) cells carrying a pET-STREP3_iPPase vector. GalKSpe4, GalK, and iPPase were purified by affinity chromatography using Strep-tag. *N*-acetylhexosamine 1-kinase from *Bifidobacterium longum* (NahK; EC 2.7.1.162; (67)) was expressed in *E. coli* BL21 (DE3) carrying a pET21a expression vector. UDP-glucose pyrophosphorylase from *B. longum* (UGPase; EC 2.7.7.9; (68)) was produced in *E. coli* BL21 (DE3) Gold cells carrying a pET30_UGPase vector. NahK and UGPase were purified by affinity chromatography using a His tag. Pyruvate kinase from rabbit muscle (EC 2.7.1.40) was purchased from Sigma–Aldrich. Full details on gene expression, affinity-tag protein purification, and activity assays are provided elsewhere (17).

### Enzyme activity assay

Purified *Ta*TyvE variants (0.2–40 mg ml^−1^; 5.1–1020 μM) and sugar nucleotides (4.0 mM) were reacted at 60 °C in MOPS buffer (100 mM, pH 7.5) and 50 mM NaCl without agitation. Enzyme solutions (45 μl) were pre-equilibrated for 5 min at 60 °C. Reactions were initiated by adding 5 μl of sugar nucleotide solution (40 mM). Samples (3.0 μl) were taken every 5 min, transferred to 30 μl deionized water, and quenched with methanol (50% v/v final concentration). Mixtures were centrifuged (21,130 *g;* 40 min), and supernatants were analyzed on CE. Initial product formation rates were calculated from linear segments of the time-course data by dividing the slope of the linear regression (mM min^−1^) by the enzyme concentration (mg ml^−1^), yielding specific activities in μmol min^−1^ (mg_protein_). One unit (U) of *Ta*TyvE activity is defined as the amount of enzyme producing 1 μmol product/min under the conditions used. Experiments were performed in triplicate (*N* = 3).

### Substrate synthesis

#### CDP–4-deoxy-α-d-glucose

The reaction (Fig. S13*A*) was performed in a 1.5 ml Eppendorf tube with a total volume of 200 μl. The reaction mixture contained GalKSpe4 (4.0 mg ml^−1^), UGPase (4.0 mg ml^−1^), iPPase (1.0 mg ml^−1^), ATP (300 mM), CTP (300 mM), MgCl_2_ (200 mM), and 4-deoxy-d-glucose (200 mM) in MOPS buffer (100 mM, pH 7.8) (69). The reaction was carried out at 37 °C for 16 h (80 rpm) on an Eppendorf thermomixer comfort, and reaction progress was analyzed by HPLC. The samples were prepared as described under “Enzyme activity assay.” The product was isolated as detailed in the “Product isolation” section. The CE chromatogram of the purified CDP–4-deoxy-Glc is presented in Figure S13*B*.

#### CDP–4-deoxy-4-fluoro-α-d-glucose

Synthesis of CDP–4-fluoro-Glc (Fig. S14*A*) was carried out in a 15 ml Sarstedt tube with a final reaction volume of 5 ml. GalKSpe4 (4.0 mg ml^−1^), pyruvate kinase (10.0 U/ml), 4-deoxy-4-fluoro-d-glucose (50 mM), ATP (1 mM), phosphoenolpyruvate (30 mM) and MgCl_2_ (6 mM) were reacted in Tris–HCl buffer (50 mM; Ph 7.5). The reaction mixture was incubated at 37 °C (20 h) without agitation, and reaction progress was monitored by TLC. CMP coupling (30 °C; 20 h) was initiated by the addition of CTP (40 mM), bovine serum albumin (1.3% w/v), UGPase (2.0 mg ml^−1^), and iPPase (0.2 mg ml^−1^) and analyzed by HPLC. Sample preparation and product isolation were performed as described in the previous section. The CE chromatogram of the isolated compound is shown in Figure S14*B*.

#### CDP–α-d-galactose

The reaction (Fig. S15*A*) was conducted in a 15 ml Sarstedt tube with a total volume of 7 ml. GalK (5.0 mg ml^−1^), ATP (20 mM), MgCl_2_ (5 mM), and d-galactose (25 mM) were incubated (20 h) at 30 °C in MOPS buffer (100 mM, pH 7.5) without agitation. Phosphorylation progress was monitored by TLC. Nucleotidyl transfer was promoted by the addition of UGPase (2.0 mg ml^−1^) and iPPase (1.0 mg ml^−1^) and analyzed by HPLC. The procedures for sample preparation and product purification are detailed under “Enzyme activity assay” and “Product isolation.” The CE chromatogram of the purified CDP–α-d-galactose is presented in Figure S15*B*.

#### CDP–α-d-lyxose

Chemical synthesis (70) of CDP–α-d-lyxose (Fig. S16*A*) was conducted by mixing 2-chloro-1,3-dimethylimidazolinium chloride (76 mg, 450 μmol) dissolved in D_2_O/CH_3_CN (2:1, 200 μl) with d-lyxose (27 mg, 150 μmol) predissolved in D_2_O/CH_3_CN (2:1, 400 μl). After adding Et_3_N (250 μl, 1.8 mmol) and CDP (105 mg, 152 μmol), the reaction mixture was stirred (800 rpm) at 4 °C for 72 h. Reaction progress was monitored by HPLC. Samples (2 μl) were diluted 1:100 in a 1:1 (v/v) mixture of deionized water and CH_3_CN. See the section “Product isolation” for work-up. The CE chromatogram and ^1^H-NMR spectrum of the purified CDP–α-d-lyxose are presented in Figures S16*B* and S17, respectively.

#### CDP–α-d-glucose

Synthesis of CDP–α-d-glucose was carried out in a 50 ml Sarstedt tube with a total volume of 15 ml. NahK (5.0 mg mL^−1^), ATP (20 mM), MgCl_2_ (5 mM), and d-glucose (25 mM) were incubated (20 h) at 30 °C in MOPS buffer (100 mM, pH 7.5) without agitation. Phosphorylation was monitored by TLC. CMP coupling was initiated by the addition of UGPase (1.0 mg ml^−1^), CTP (25 mM), and iPPase (2.0 mg ml^−1^). Reaction progress was analyzed by HPLC. The procedures for sample preparation and product purification are described under “Enzyme activity assay” and “Product isolation.” The reaction scheme, HPLC chromatogram, and ^1^H-NMR spectrum are provided in Ref. (17).

### Product isolation

Enzymes were removed by ultrafiltration using Vivaspin centrifugal tubes (10 kDa cutoff; Sartorius) prior to product isolation. Separation of sugar nucleotides from other components was accomplished by anion exchange chromatography (125 ml Toyopearl SuperQ-650M column; Tosoh Bioscience) using an ÄKTA FPLC system (GE Healthcare). Sodium acetate buffers (20 mM and 1 M; pH 4.3) were used for binding and elution of compounds by a step-wise gradient. Product-containing fractions were identified based on UV absorbance at 254 nm, collected, and concentrated under reduced pressure (20 mbar, 40 °C) using a rotary evaporator (Laborota 4000; Heidolph). Removal of sodium acetate was achieved in a Sephadex G-10 size-exclusion column (GE Healthcare) using deionized water as the eluent. Fractions containing the target product were concentrated under reduced pressure and freeze-dried on a Christ Alpha 1–4 lyophilizer (B. Braun Biotech International). Full details of all procedures are provided elsewhere (17).

### Gel filtration

A HiLoad 16/600 Superdex 200 pg column connected to an Äkta go chromatography system from Cytiva was used for gel filtration. A running buffer containing 10 mM Hepes, 150 mM NaCl, and 0.1 mM Tris(2-carboxyethyl)phosphine (pH 7.5) was used at a flow rate of 1 ml min^−1^. To assess potential oligomeric states and concentration-dependent shifts in elution behavior, gel filtration was performed with three different concentrations of HisTag-purified *Ta*TyvE (Fig. S3*B*). Samples at 0.5, 1.0, and 2.0 mg ml^−1^ were prepared in running buffer and injected onto the column at a constant injection volume of 500 μl. All chromatographic runs were performed under identical conditions to ensure comparability between injections. The column was calibrated with the gel filtration standard mixture #1511901 (Bio-Rad Laboratories, Inc). Logarithmic molecular masses of thyroglobulin (670,000 Da), bovine γ-globulin (158,000 Da), chicken ovalbumin (44,000 Da), equine myoglobin (17,000 Da), and vitamin B12 (1350 Da) were plotted against their respective elution volumes (Fig. S3*A*). The protein standard curve was used to provide an estimate of the molecular mass of *Ta*TyvE.

### Crystallization

Prior to crystallization, His-tag-purified *Ta*TyvE (wildtype) was subjected to gel filtration (see Gel filtration section) for removal of protein aggregates. Crystallization plates were set up with an ORYX 8 pipetting robot (Douglas Instruments) and commercially available Index and SG1 (ShotGun) screens at 20 °C *via* the sitting drop method. Drops were dispensed in a 1:1 ratio (0.5 μl precipitant/0.5 μl protein). The protein solution contained 1 mM NAD^+^, 1 mM CDP, and 7.5 to 30 mg ml^−1^ *Ta*TyvE. Well-diffracting crystals were observed after 7 days using 0.2 M potassium thiocyanate, 20% PEG 3350 (ShotGun D4), and 7.5 mg ml^−1^ of *Ta*TyvE. Harvested crystals supplemented with 15% glycerol were flash-frozen in liquid nitrogen prior to X-ray data collection at the P13 beamline operated by EMBL Hamburg at the PETRA III storage ring (DESY). Data were collected at a resolution of 2.6 Å and processed with XDS (Wolfgang Kabsch) (71). The *Ta*TyvE crystal structure (PDB ID: 9RL0) was solved by molecular replacement using the *St*TyvE (65% sequence identity) structure complexed with CDP and NAD-coenzyme (PDB ID: 1ORR) as a template. The model was rebuilt and refined using PHENIX (72) and Coot (73). The structure was validated and analyzed with MolProbity (all-atom contact analysis) and PDBePISA (analysis of interfaces and protomer assembly) (46, 74), (http://www.ebi.ac.uk/pdbe/prot_int/pistart.html).

### Structural depictions and molecular docking

PyMOL Molecular Graphics System 2.5.4 (Open-Source, Schrödinger, LLC) and UCSF ChimeraX (75) were used for depiction of protein structures. Molecular docking was performed using Yasara (Yasara Biosciences GmbH) and Autodock Vina (76). The Amber15FB force field involving bond-, angle-, dihedral-, planarity-, Coulomb-, and van der Waals–force field terms was applied. Limits of nonbonded short-range interactions were set to 10.5 Å. Residues of the enzyme–NAD^+^ receptor molecule were designated as flexible. The simulation cell was positioned around the *Ta*TyvE active site. Ligands (CDP–Glc, CDP–Man) were designed in ChemDraw (PerkinElmer Informatics, Inc) and treated as flexible during global docking to the enzyme–NAD^+^ receptor. The generated enzyme–substrate complexes served as input structures for MD simulations.

### MD simulations

Both substrates, CDP–Glc and CDP–Man, were simulated *via* classical MD simulations bound to the tetrameric *Ta*TyvE in the presence of NAD^+^ as a coenzyme. The force field parameters of Amber19SB and GLYCAM06j-1 were used to represent the protein residues as well as the sugar moieties in the system. NAD^+^ was represented using Ryde's parameters (77) available at the AMBER parameter database (http://amber.manchester.ac.uk/). The geometries of CDP–Man and CDP–Glc were optimized with BP86/def2-SVP using an implicit solvation model for water, and the RESP charges (net charge −2 for both of them) were calculated afterward with antechamber from an electrostatic potential calculated at the level of theory of HF/6-31G∗. All quantum mechanical calculations were done using Gaussian16. AMBER atom types were used to represent the substrate atoms. The protonation state at pH 7.5 of all titratable residues in T*a*TyvE was calculated using the server H++ (78). Both substrates were placed at the active site *via* molecular docking (see the Structural depictions and molecular docking section). The two substrate–enzyme complexes were embedded into a box of OPC water molecules and neutralized with 8 Na^+^ atoms. Before the production phase, the two solvated systems were minimized, heated up to 333 K, and equilibrated into the final simulation conditions. The minimization was done in three consecutive steps, where the geometry of all hydrogens, all molecules except the solute, and the whole system were allowed to change. The heating step was performed from 100 to 333 K in 200 ps at constant volume (NVT ensemble) using the Langevin thermostat (friction coefficient of 1 ps^−1^). During this step, the position of all solute atoms was constrained with a harmonic force of 200 kcal mol^-1^ Å^−2^. The resulting solutions were then equilibrated in five steps, during which the constraints were gradually removed. In the last equilibration step, the system was equilibrated at constant pressure (NPT ensemble). The SHAKE algorithm was applied to all covalent bonds involving hydrogens, and the electrostatic interactions in the long range were calculated using PME. Then, each system was further simulated for 500 ns per simulation without any constraint. Three independent simulations were run for each system, yielding a total simulation time of 1.5 μs per complex. An additional set of MD simulations with CDP–Glc or CDP–Man was performed using the same protocol but with the CDP moieties initialized in the conformation observed in the crystal structure of *St*TyvE (PDB ID: 1ORR; protomer D). Five independent simulations were run for each system. The analysis of all trajectories was performed using cpptraj. See Figures S9–S11 in the supporting information for average heavy-atom distances, ring pucker changes, dihedral angle distributions, and energy profiles.

### Analytical procedures

#### HPLC

Formation of sugar nucleotides from sugar 1-phosphates and nucleotides was monitored using a Shimadzu Prominence HPLC-UV system equipped with a Kinetex C18 analytical HPLC column (150 × 4.6 mm, 5 μm EVO C18 100 Å; Phenomenex). The mobile phase consisted of 20 mM potassium phosphate buffer (pH 5.9) supplemented with 40 mM tetrabutylammonium bromide (98%; solvent A) and methanol (2%; solvent B). Compounds were eluted by isocratic elution (1 ml min^−1^; 40 °C). UV detection was set to λ = 271 nm.

#### Capillary zone electrophoresis

Samples from enzyme activity assays and isolated sugar nucleotides were assessed using a 3D CE system from Hewlett Packard equipped with a diode array detector (λ = 271 nm) and an extended light path fused silica capillary (5.6 mm × 56 cm) from Agilent Technologies. Samples were loaded by pressure injection (50 mbar, 5 s or 10 s) and separated with a voltage of up to 30 kV for 12 min at 50 °C using sodium tetraborate buffer (20 mM; pH 9.3). See Figures S13–S16 in the supporting information for CE chromatograms of isolated compounds.

#### TLC

TLC was used for monitoring anomeric phosphorylation through the separation of sugars and sugar 1-phosphates. Reaction samples were analyzed on silica gel plates (gel 60 F_254_, Merck). The eluent consisted of 1-butanol/acetic acid/deionized water in a 2:1:1 (v/v/v) ratio. Visualization was performed using a staining solution consisting of thymol/ethanol/H_2_SO_4_ in a 0.5:95:5 (w/v/v) ratio.

#### ^1^H-NMR analysis

Acquisitions were carried out on a Bruker AVANCE III 300-MHz spectrometer (Bruker) with an autosampler. The Bruker Topspin 3.5 software was used for the measurements. All spectra were analyzed using MestReNova 16.0 (Mestrelab Research, S.L.).

#### Native mass spectrometry

Enzyme samples (50 pmol of *Ta*TyvE; 5 μl at 10 μM concentration) were desalted on an AdvanceBio rSEC 300 Å (2.7 μm, 4.6 × 50 mm) size-exclusion HPLC column equilibrated in 150 mM ammonium acetate (pH 7). A Shimadzu Nexera UHPLC system was used for automated injections and desalting *via* the column at a flow rate of 0.2 ml/min. The eluting proteins were directly injected into a Bruker maxis II ultra–high-resolution Q-TOF device, and the elution volumes containing salts were diverted to the waste valve. Mass spectra were acquired in an *m/z* range between 500 and 10,000 with a 2.5 bar nebulizer pressure and 8 l/min dry gas setting. The source temperature was set to 325 °C, and the collision-induced dissociation energy was set to 100 eV. Transfer time and prepulse storage were optimized for native mass spectrometry with 180 μs and 40 μs, respectively. Calibration of the mass spectrometer in the *m/z* range from 1822 to 5460 was performed using the ESI-tune mix at elevated concentrations, allowing assignments of dimeric and trimeric species. Data were analyzed using DataAnalysis (Bruker) and maximum entropy deconvolution of the protein signals. Spectra are provided in the supporting information (Fig. S4).

## Data availability

The crystal structure was deposited in the PDB (PDB ID: 9RL0). The topology and coordinates of *Ta*TyvE in complex with CDP–Glc and CDP–Man (AMBER top and crd files), the trajectories for the simulations (as dcd files), as well as the analysis files from the trajectories can be accessed at Zenodo (https://doi.org/10.5281/zenodo.17579641). All other data are contained within the article and the supporting information or will be provided upon reasonable request.

## Supporting information

This article contains supporting information (Table S1, Figs. S1–S18, discussion related to Fig. S16, and Refs. (67, 68), http://www.ebi.ac.uk/pdbe/prot_int/pistart.html, (70, 79, 80, 81, 82)).

## Conflict of interest

The authors declare that they have no conflicts of interest with the contents of this article.
