# Supplementary material for: A dual catalytic architecture promotes C-2 stereoinversion of CDP–glucose by the CDP–tyvelose 2-epimerase from Thermodesulfatator atlanticus
Source: J Biol Chem. 2026 Mar 19;302(5):111384. doi: 10.1016/j.jbc.2026.111384 (PMC13098431; doi:10.1016/j.jbc.2026.111384)
Supplement: Supporting Information [file mmc1.pdf]

Supporting Information

A dual catalytic architecture promotes C-2 stereo-inversion of CDP-glucose by the CDP-tyvelose 2-epimerase from *Thermodesulfatator atlanticus*

Christian Rapp[a], Stevie van Overtveldt[b], Pedro A. Sánchez-Murcia[c], Martin Pfeiffer[a], Koen Beerens[b], Magdalena Merkaš[d], Tea Pavkov-Keller[e], Tom Desmet[b,f] and Bernd Nidetzky[a,f,#]

[a] Institute of Biotechnology and Biochemical Engineering, Graz University of Technology, NAWI Graz, 8010 Graz, Austria

[b] Centre for Synthetic Biology, Department of Biotechnology, Ghent University, 9000 Ghent, Belgium

[c] Laboratory of Computer-Aided Molecular Design, Division of Medicinal Chemistry, Otto-Loewi Research Center, Medical University of Graz, BioTechMed-Graz, 8010 Graz, Austria

[d] Institute of Molecular Biotechnology, Graz University of Technology, NAWI Graz, 8010 Graz, Austria

[e] Institute of Molecular Biosciences, University of Graz, 8010 Graz, Austria

[f] Austrian Centre of Industrial Biotechnology (acib), 8010 Graz, Austria

# Corresponding author: bernd.nidetzky@tugraz.at

Table S1. Data collection and refinement statistics for the *Ta*TyvE crystal structure with bound NAD^+ and CDP. Statistics for the highest-resolution shell are shown in parentheses.

Wavelength (Å)	1.072
Resolution range (Å)	46.6 - 2.6 (2.63 - 2.6)
Space group	$P\ 2_1$
Unit cell (Å, °)	132.42, 90.51, 133.62<br>90 ,101.19, 90
Total reflections	301130 (5013)
Unique reflections	174040 (3388)
Multiplicity	1.7 (1.5)
Completeness (%)	97.6 (68.7)
Mean I/sigma(I)	4.06 (0.85)
Wilson B-factor	44.04
R-merge	0.129 (0.804)
R-meas	0.179 (1.12)
R-pim	0.124 (0.773)
CC1/2	0.98 (0.43)
CC*	0.99 (0.78)
Reflections used in refinement	93324 (2174)
Reflections used for R-free	4666 (109)
R-work	0.242 (0.321)
R-free	0.290 (0.368)
Number of non-hydrogen atoms	22362
macromolecules	21952
ligands	402
solvent	8
Protein residues	2688
RMS(bonds)	0.004
RMS(angles)	0.63
Ramachandran favored (%)	96.09
Ramachandran allowed (%)	3.80
Ramachandran outliers (%)	0.11
Rotamer outliers (%)	1.53
Clashscore	6.64
Average B-factor	48.73
macromolecules	48.83
ligands	43.48
solvent	42.49

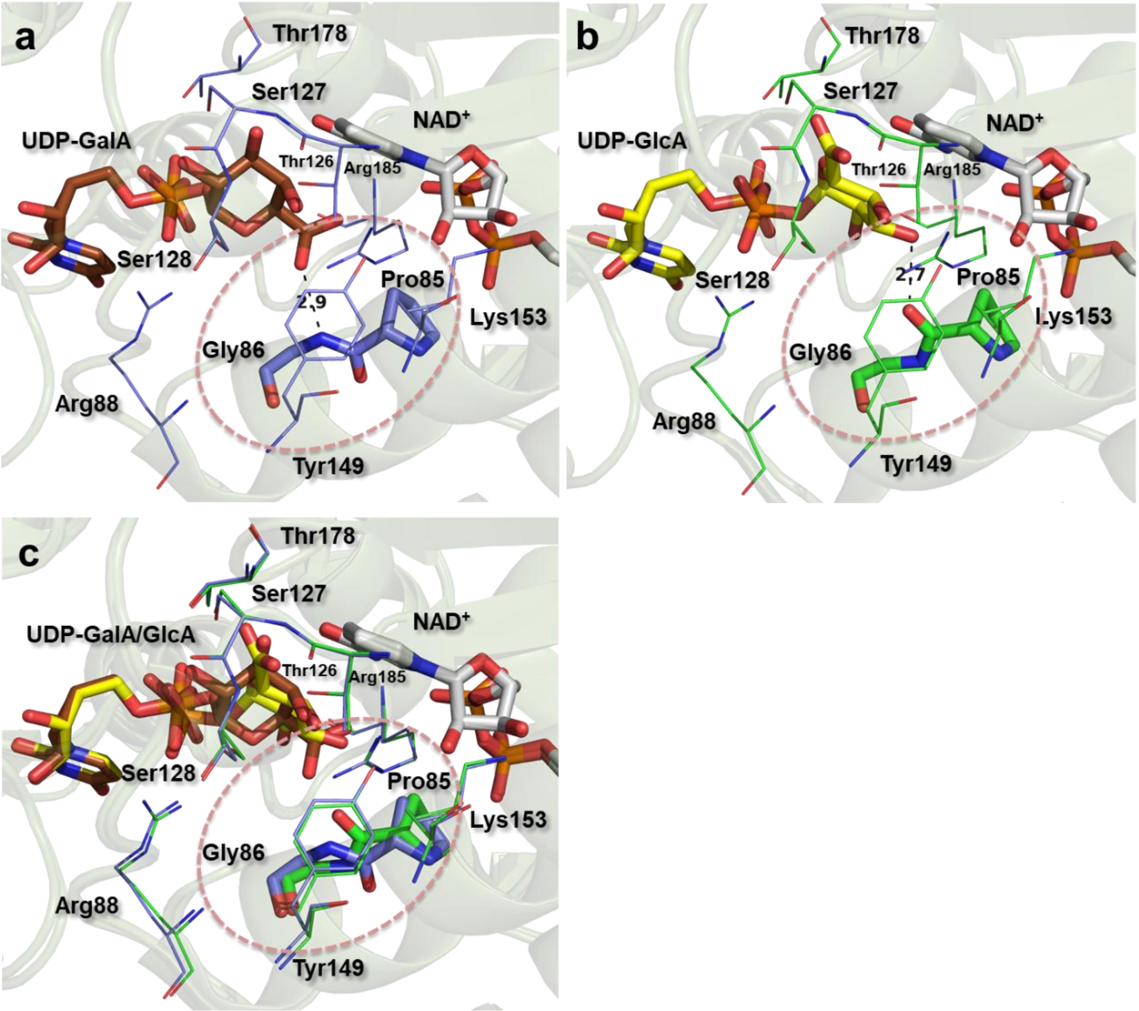

Figure S1. Active site of GlcAE from *Bacillus cereus* with bound coenzyme and sugar nucleotide epimers. GlcAE co-crystallized with NAD^+ and (**a**) UDP-galacturonic acid (PDB: 6ZLL) or (**b**) UDP-glucuronic acid (PDB: 6ZLD). Interaction of the Gly86 main chain-NH with the sugar carboxylate and the Pro85 main chain carbonyl with sugar C3-OH are explicitly shown (salmon dotted circle). (**c**) Structural overlay of **a** and **b** depicting the Pro85-Gly86 dipeptide flip within an overall rigid active site. Color code: NAD^+: grey; UDP-glucuronic acid: yellow; UDP-galacturonic acid: brown.

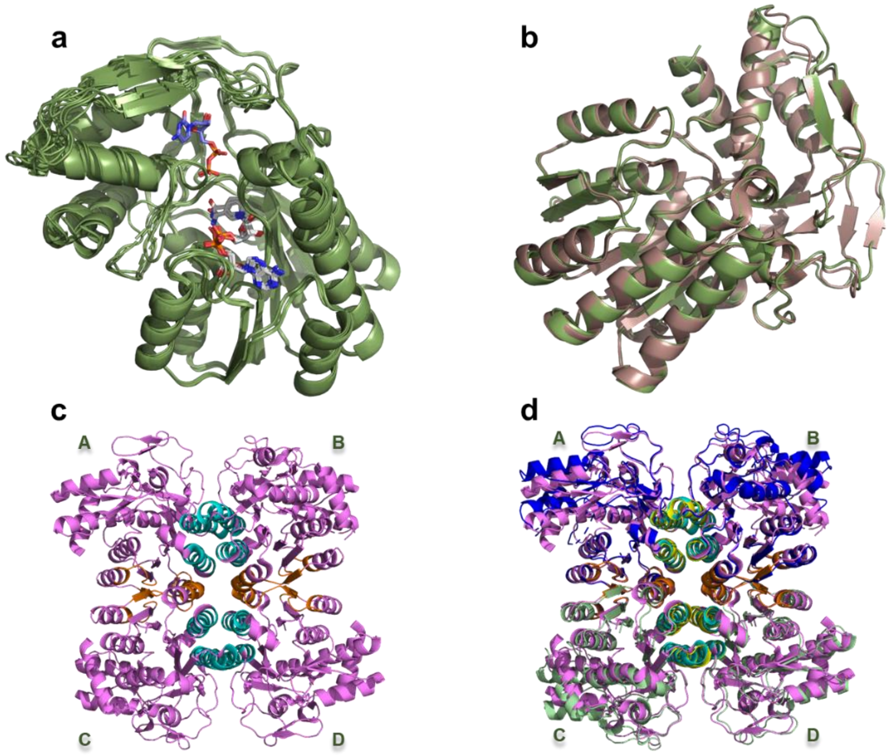

Figure S2. Comparison of tertiary and quaternary structure of *Ta*TyvE and *St*TyvE. (**a**) Superposition of all *Ta*TyvE protomers. CDP and NAD^+ are depicted in blue and grey, respectively. (**b**) Structural overlay of *Ta*TyvE protomer A (green) and *St*TyvE protomer A (salmon). (**c**) Quaternary structure of *St*TyvE (PBD: 1ORR) showing protomers A – D and subunit interactions (cyan, orange). (**d**) Overlay of *Ta*TyvE and *St*TyvE quaternary structures.

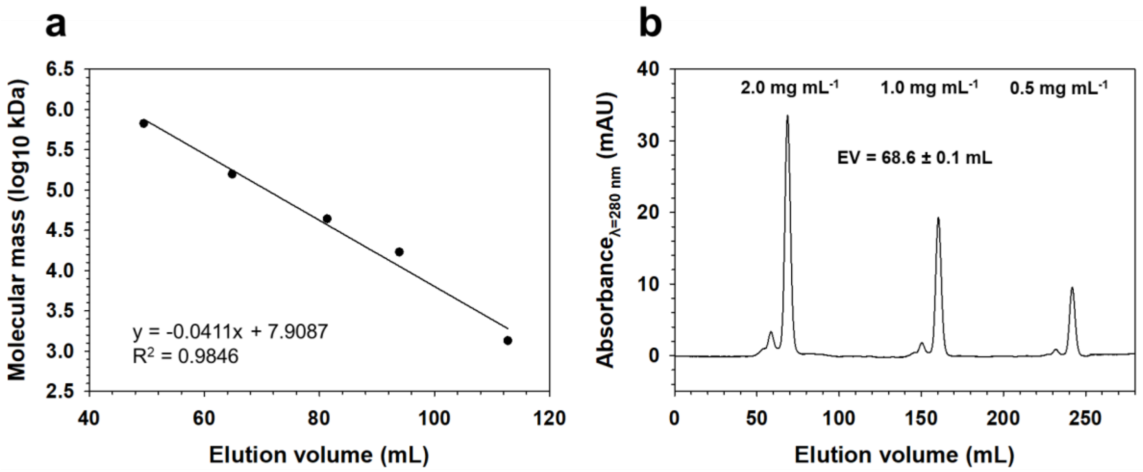

Figure S3: Elution behavior of *Ta*TyvE across different protein concentrations. (**a**) Calibration curve and linear regression generated using protein standards of known molecular mass. The corresponding equation is shown in the inset. (**b**) Gel-filtration profiles of *Ta*TyvE at varying concentrations (0.5-2.0 mg mL^{-1}). The elution volume (EV) nominally corresponds to an apparent trimeric assembly.

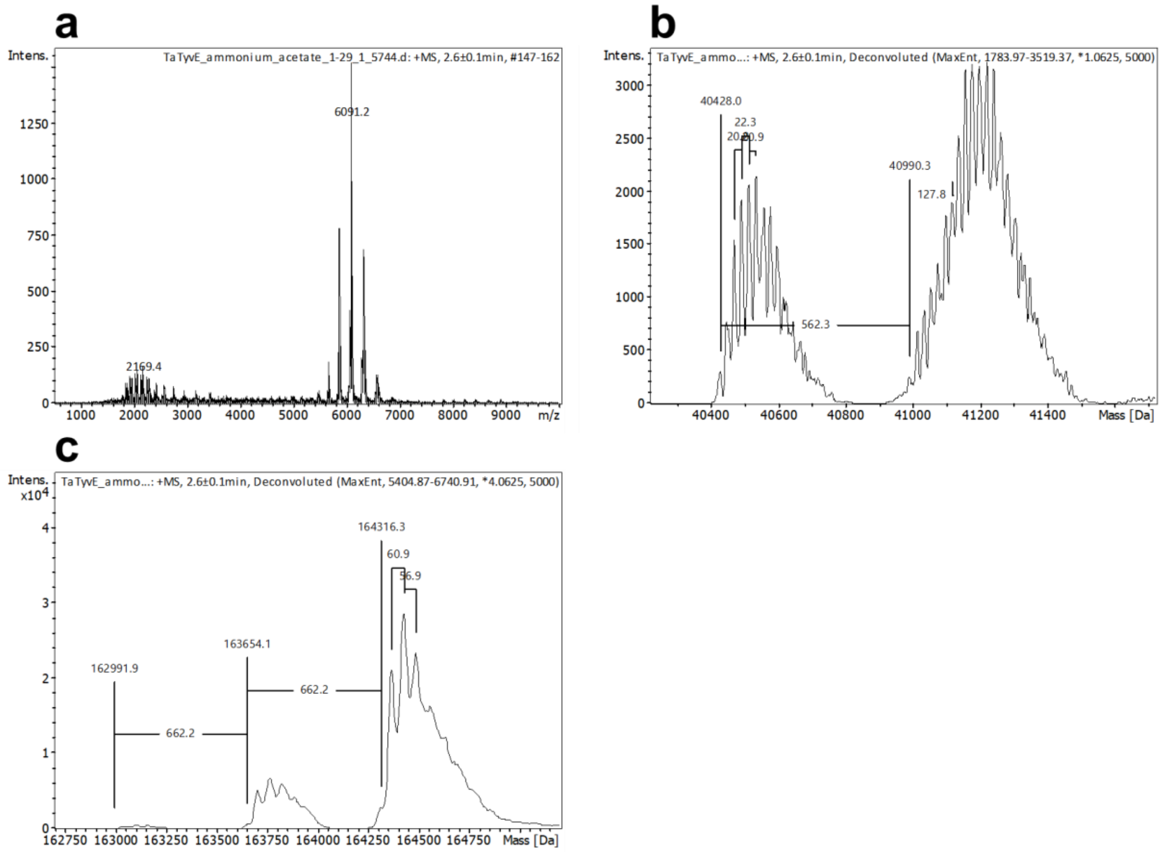

Figure S4. Native mass spectra of *Ta*TyvE. (**a**) Raw spectrum showing *Ta*TyvE predominantly as tetramer, with three distinct acetate adducts. (**b**) Deconvoluted spectrum of the *Ta*TyvE protomer. The expected molecular mass of the His-tagged protomer without bound coenzyme is 40144.11 Da. Three sodium adducts are observed. Additional mass differences of ~562 Da likely originate from bound nucleotide sugars retained from *E. coli* expression. (**c**) Deconvoluted spectrum of the *Ta*TyvE tetramer. Observed mass differences of ~663 Da are consistent with bound NAD^+.

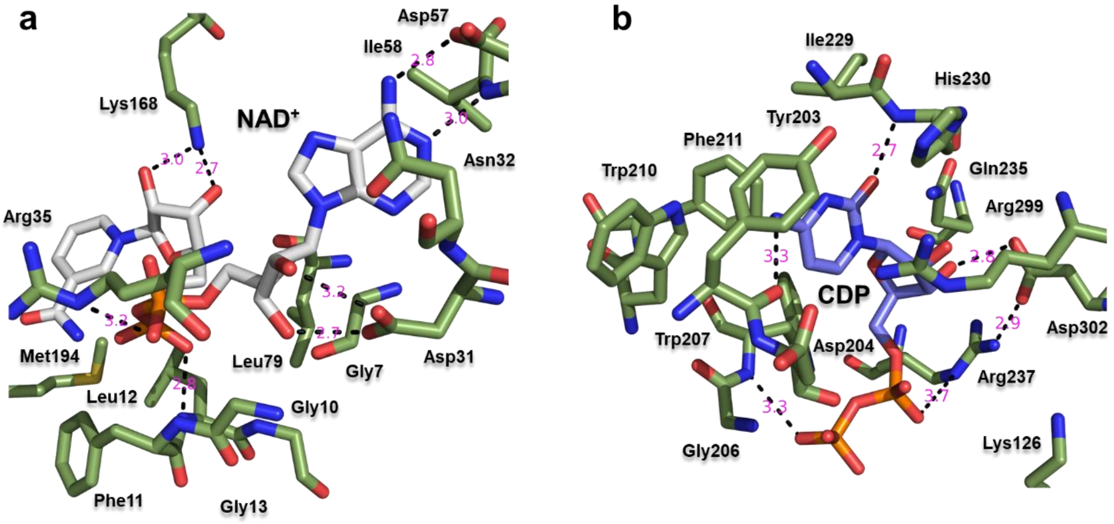

Figure S5. Binding sites for NAD^+ and CDP in the *Ta*TyvE crystal structure. Residues involved in accommodating NAD^+-coenzyme (**a**) and CDP-nucleotide (**b**) are structurally well conserved across all eight protomers. Note that the measured heavy-atom distances reflect non-covalent, short-range electrostatic interactions, including hydrogen bonds as well as hydrogen-bond-like and salt-bridge-like contacts, thereby illustrating a dynamic interaction network rather than static donor-acceptor pairings.

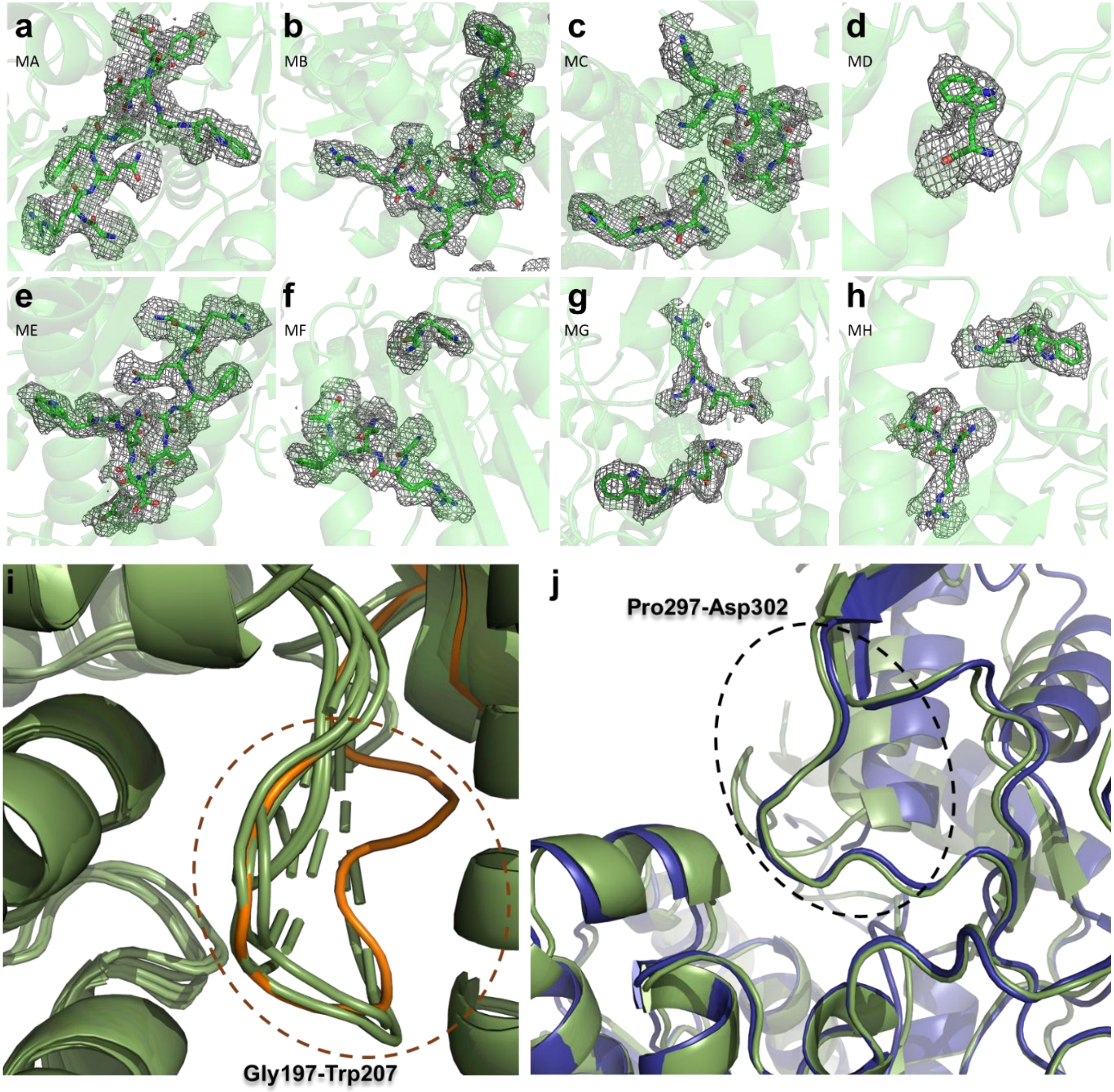

Figure S6. Loop regions Gly197 to Trp207 and Pro297 to Asp302 in *Ta*TyvE. (**a-h**) 2F_o - F_c electron density maps contoured at 1σ showing the Gly197 to Trp207 loop region in protomers A – H (Mi; M = protomer, i = A – H). In protomers A, B and E, continuous electron density allowed modeling of the entire loop. (**i**) Structural overlay of Gly197 to Trp207 loop regions from protomers B – D and F – H lacking bound CDP, with protomer B depicted in orange. (**j**) Loop region Pro297 to Asp302 (black dotted circle) of protomer F adopts a conformation (blue) as observed in *Ta*TyvE with bound CDP (green).

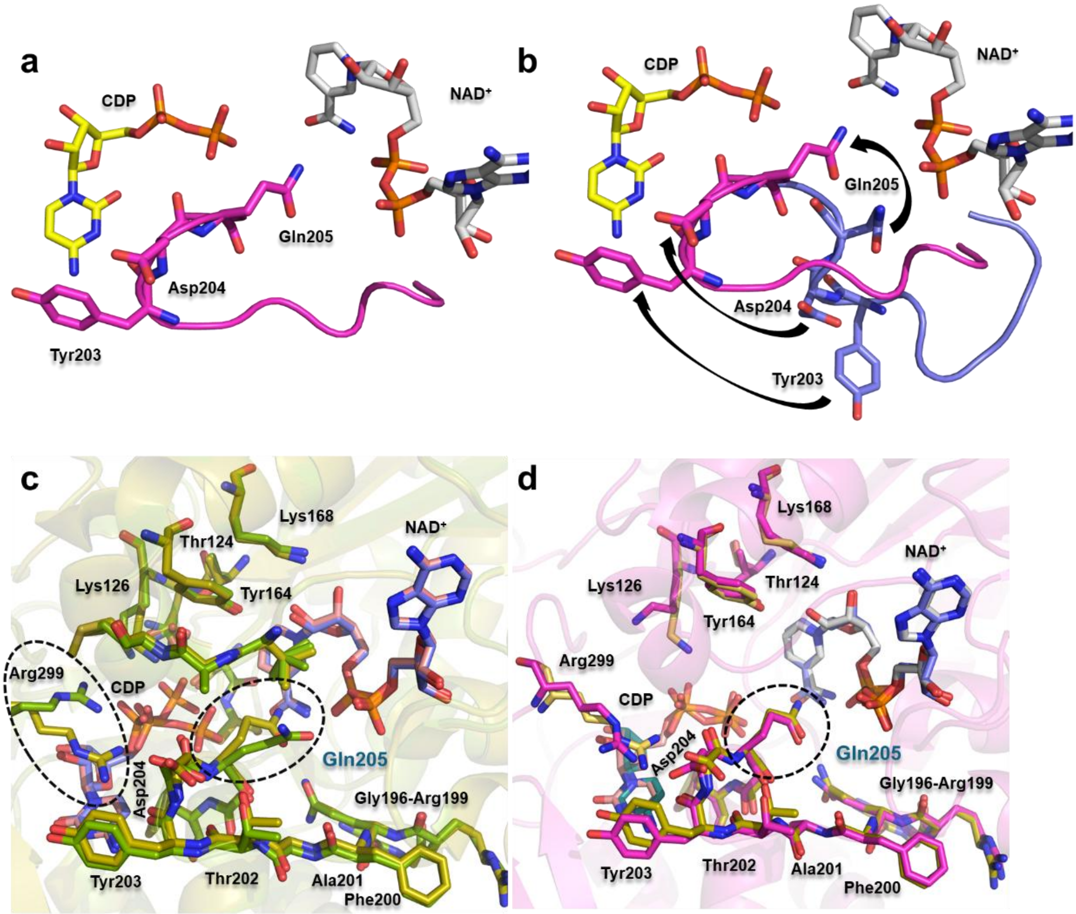

Figure S7. Open- and closed-loop conformations in *Ta*TyvE and *St*TyvE. (**a**) Ordered conformation of the Gly197-Trp207 loop in protomer A of *Ta*TyvE. (**b**) Structural overlay of protomer A (pink) and protomer B (blue) of *Ta*TyvE. Positional shifts of Tyr203, Asp204 and Gln205 upon CDP binding are highlighted. (**c**) Superposition of *St*TyvE protomers A (green residues) and D (yellow residues). Changes in the rotameric state of the Gln205 side chain as well as Arg299 are highlighted (dotted circle). (**d**) *Ta*TyvE protomer A and *St*TyvE protomer D superposed, with Gln205 framed and comparable CDP accommodation. *Ta*TyvE-residues: pink; NAD^+: grey; CDP: salmon. *St*TyvE-residues: yellow; NAD^+: blue; CDP: turquoise.

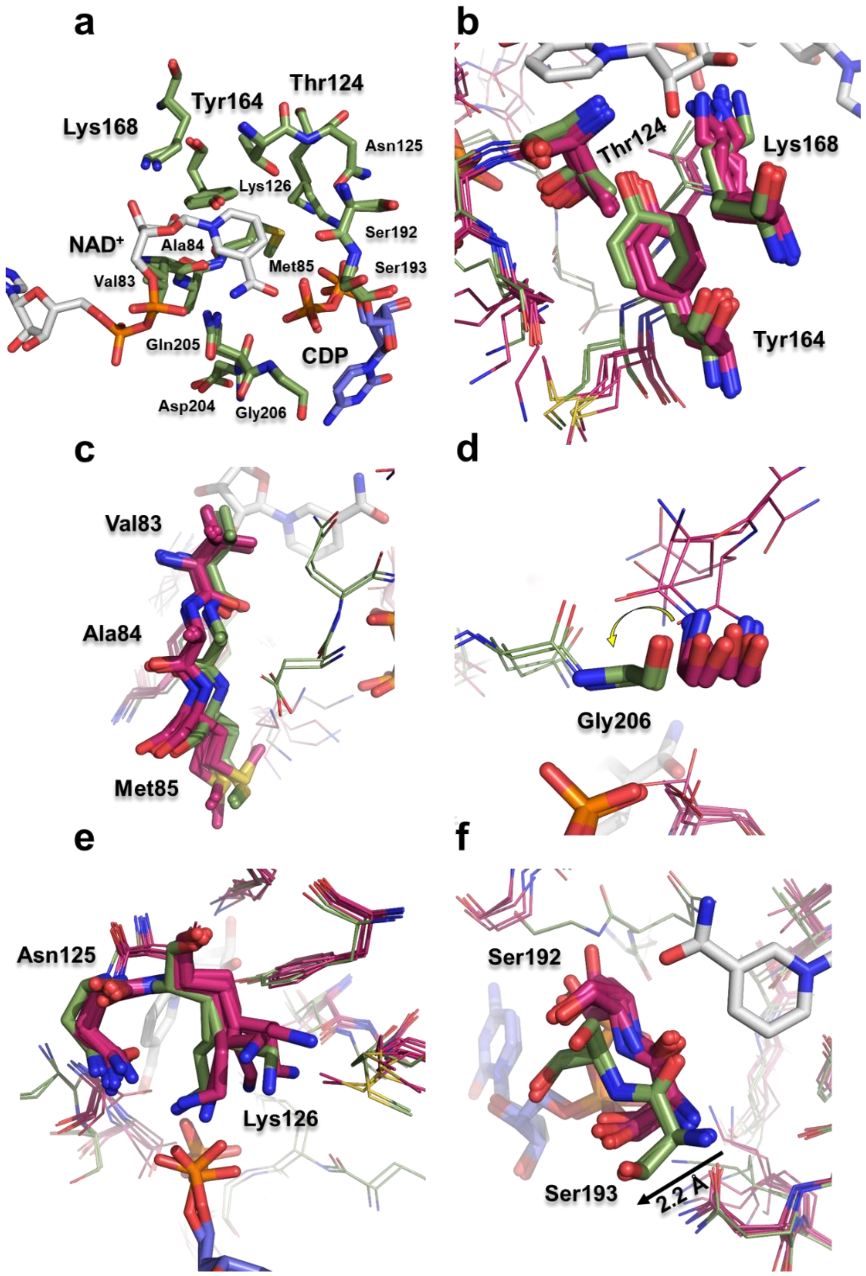

Figure S8. Putative sugar binding residues in the *Ta*TyvE active site. (**a**) Overlay of ternary structures (protomers A, E; green residues) with both CDP and NAD^+ bound. Except for the Lys126 side chain, residues are superimposable. Structural overlays of all (=8) *Ta*TyvE protomers are shown in panels **b** – **f**. Putative sugar binding residues are labeled and depicted as sticks in the respective panel. (**b**) The catalytic triad remains structurally conserved. (**c**) Main chains of Val83, Ala84 and Met85 are rigid, with minimal displacement of 0.9 Å. (**d**) The Gly206 main chain-NH is flipped in the ternary complex (yellow arrow). (**e**) Main chains of Asn125 and Lys126 retain high rigidity. (**f**) Main chains of S192 and S193 are displaced by ~2.2 Å (black arrow). For positioning of Asp204 and Gln205 in the closed loop see **Figure S7**.

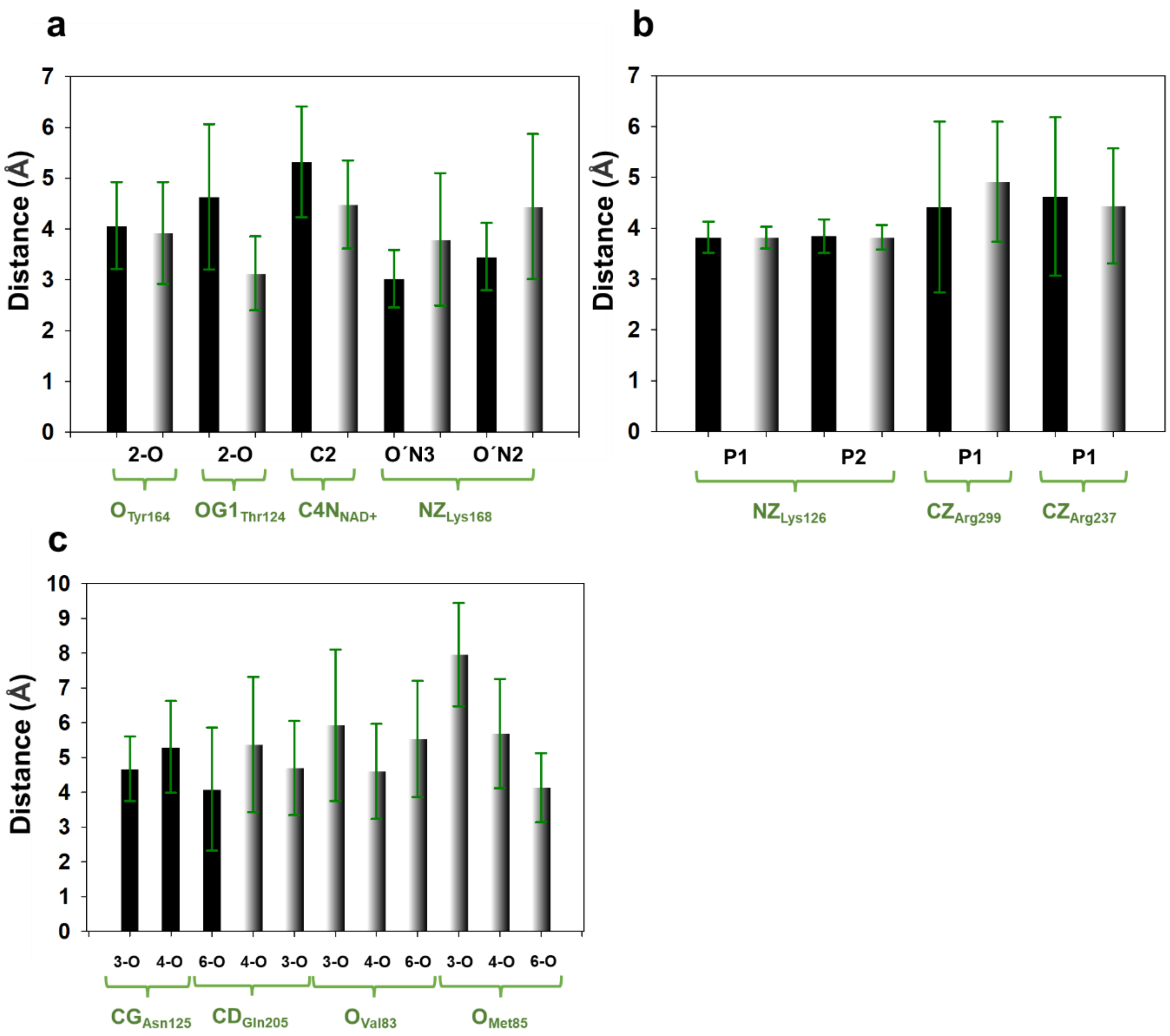

Figure S9. Heavy-atom distances of CDP-Glc and CDP-Man throughout the MD simulations within the *Ta*TyvE active site. Distances involving (**a**) the catalytic triad, (**b**) the pyrophosphate and (**c**) oxygens of non-reactive hydroxyl groups of the sugar moiety are shown. Residues proximal to CDP-Glc (black bars) and CDP-Man (grey bars), along with their corresponding distances, are displayed below the x-axis.

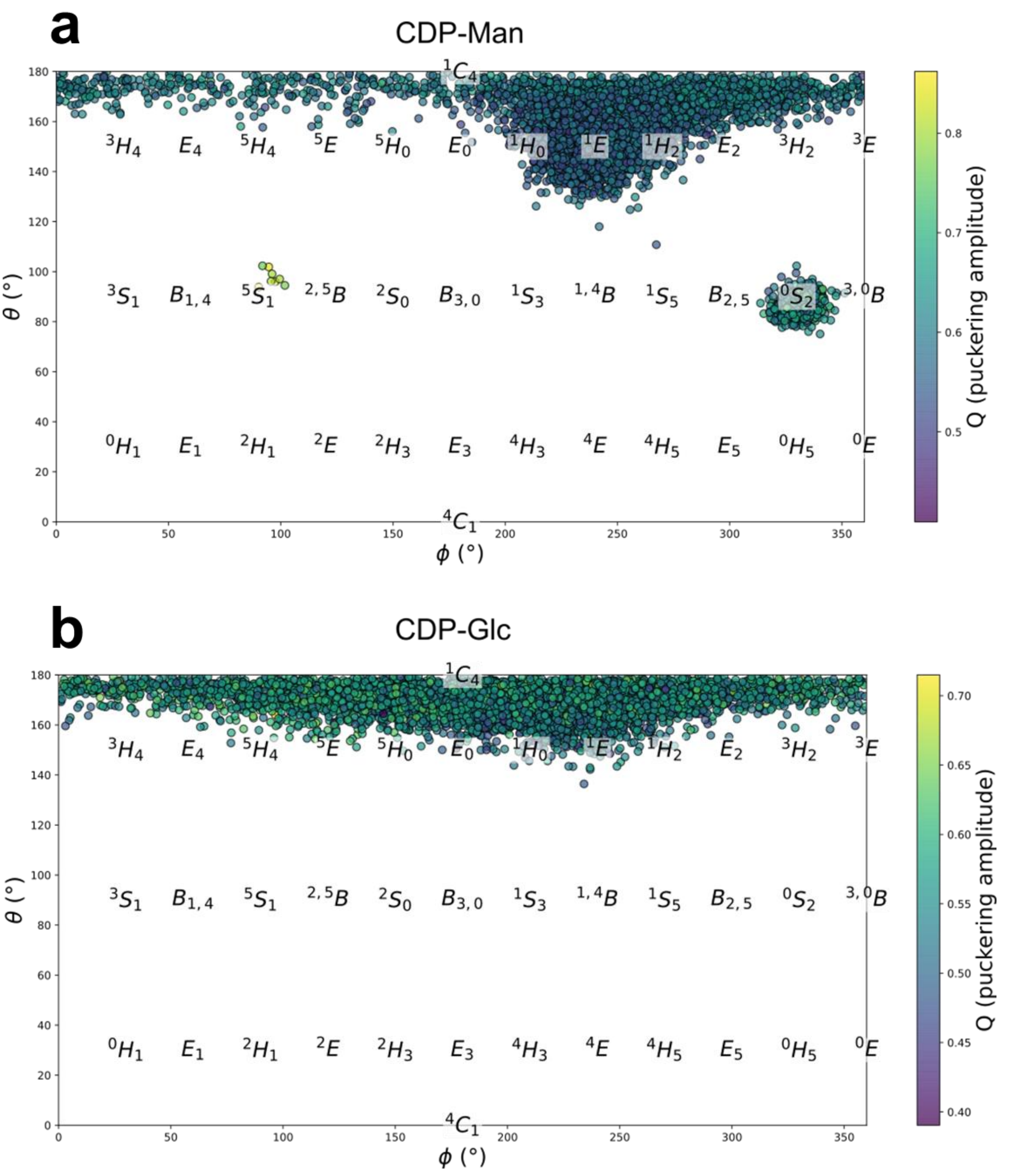

Figure S10: Cremer-Pople plot of the hexopyranosyl moieties of CDP-Man and CDP-Glc extracted from MD simulations of the enzyme-bound complexes. Ring puckering sampled by (**a**) mannose and (**b**) glucose within the active site is shown. Note: Due to the applied force field parameters, $^4C_1$ chair conformations are generally most prevalent.

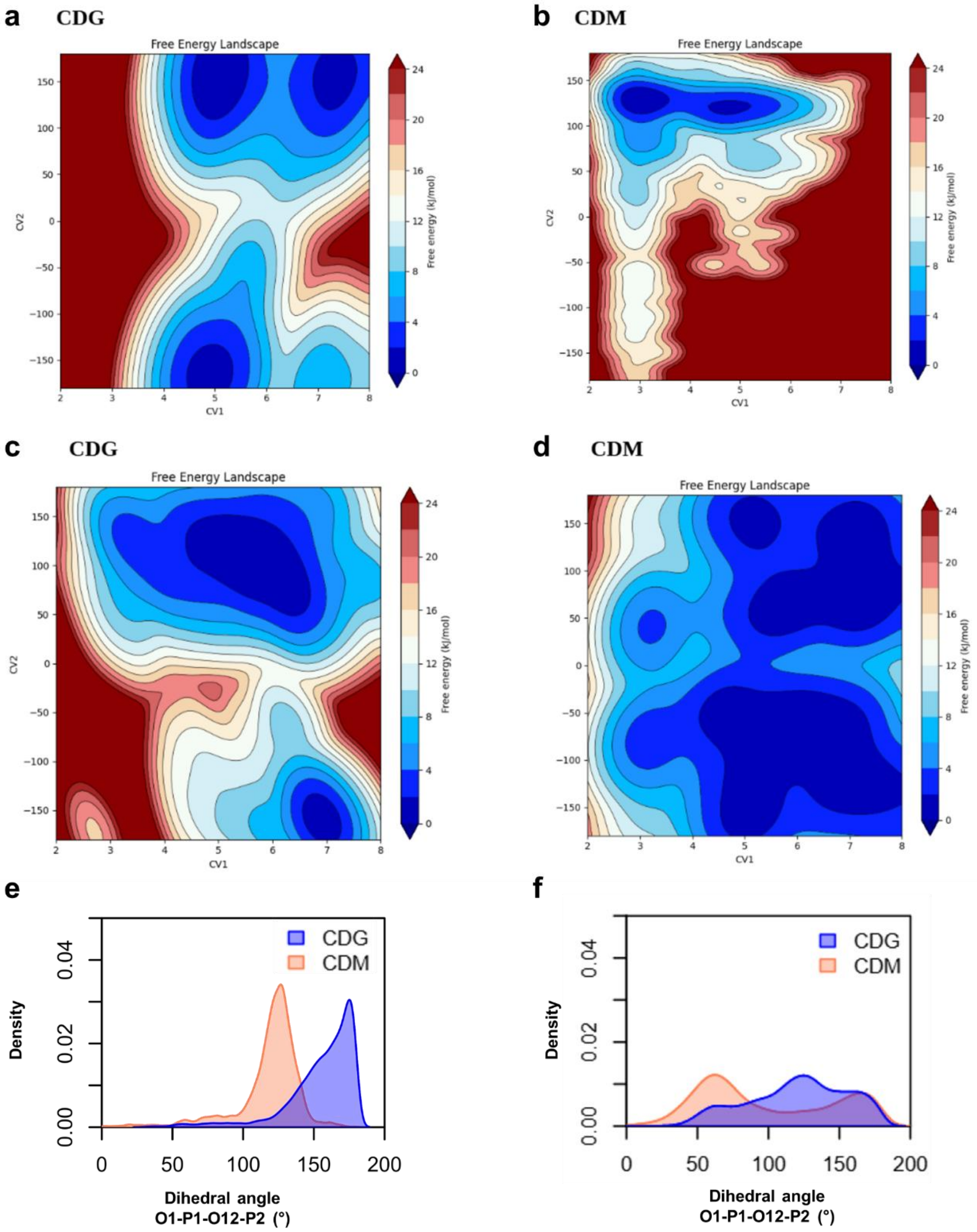

Figure S11: Comparison of energy profiles obtained from MD simulations of CDP-Man and CDP-Glc bound to *Ta*TyvE. Starting conformations were derived from docking solutions or CDP moiety accommodations in the *St*TyvE crystal structure (PDB: 1ORR). Heatmaps across CV1 and CV2 are shown for docking-derived (**a**,**b**) and *St*TyvE-based (**c**,**d**) CDP orientations of CDP-Glc (CDG) and CDP-Man (CDM). CV1: Dihedral angle O1-P1-O12-P2 (°); CV2: Distance between Tyr164-O and sugar C2-O (Å). Blue and red denote low- and high-energy regions, respectively. Energy profiles were generated using the *Free energy landscape analysis* tool. Corresponding density of the pyrophosphate dihedral angle in CDG (blue) or CDM (salmon) from either (**e**) the docking- or (**f**) *St*TyvE-derived CDP pose. Dihedral angles are reported as absolute values.

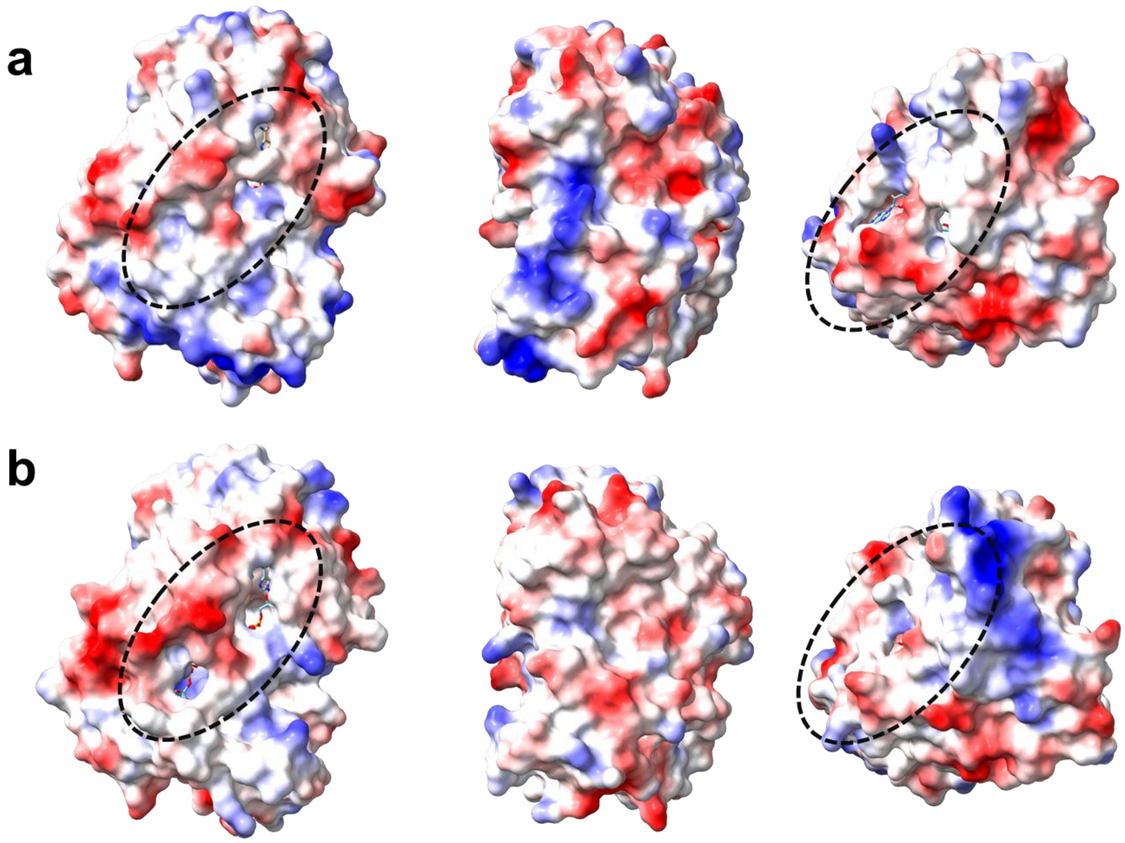

Figure S12. Electrostatic surface representations of protomer A from the crystal structures of *St*TyvE and *Ta*TyvE. (**a**) *St*TyvE is shown in three distinct orientations. (**b**) *Ta*TyvE is displayed in the corresponding orientations as in panel **a**, following structural superposition of the two enzymes to enable direct comparison. Electrostatic potentials are color-coded as follows: red: negative potential; blue: positive potential; white/gray: neutral regions. Binding pockets are framed (dotted circle).

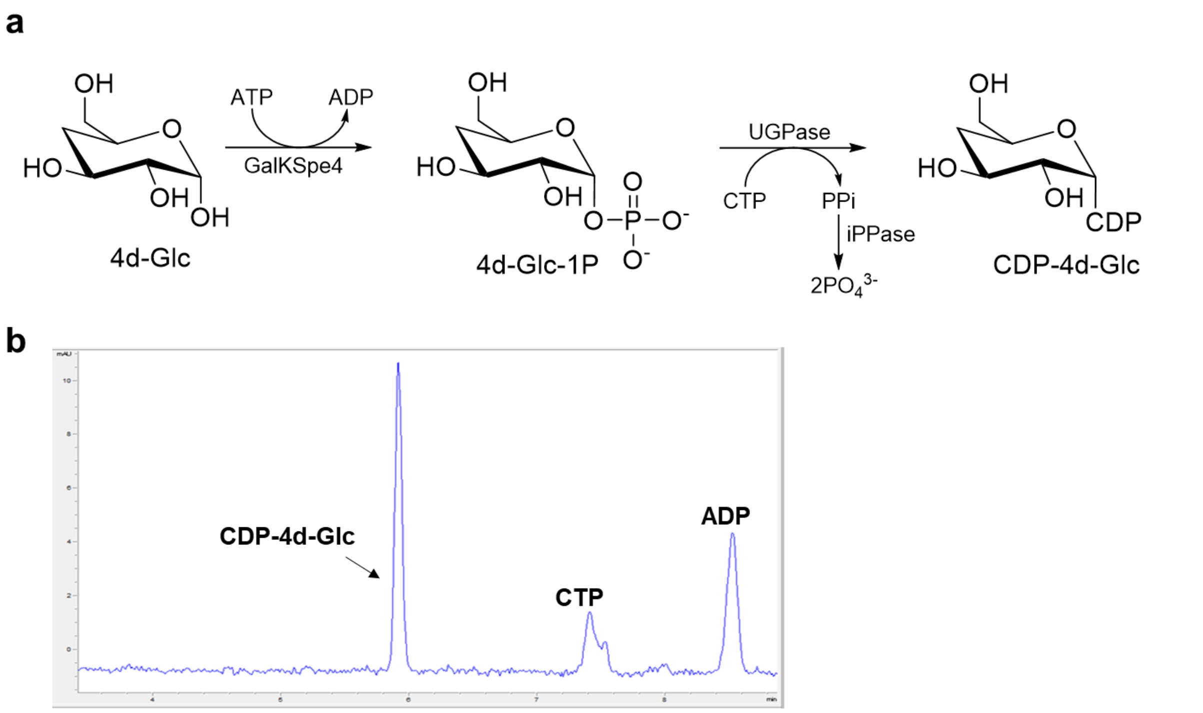

Figure S13. Synthesis of CDP-4-deoxy-α-D-glucose (CDP-4d-Glc) via a one-pot enzymatic cascade reaction and CE chromatogram of the purified compound. (**a**) GalKSpe4, UGPase and iPPase were reacted with ATP, CTP, $MgCl_2$ and 4-deoxy-D-glucose (4d-Glc). The reaction proceeds through the formation of 4-deoxy-D-glucose-1-phosphate (4d-Glc-1P) as an intermediate en route to CDP-4d-Glc. (**b**) CE chromatogram of isolated CDP-4d-Glc showing CTP (UV_{271nm} ~12%) and ADP (UV_{271nm} ~32%) as the main contaminants.

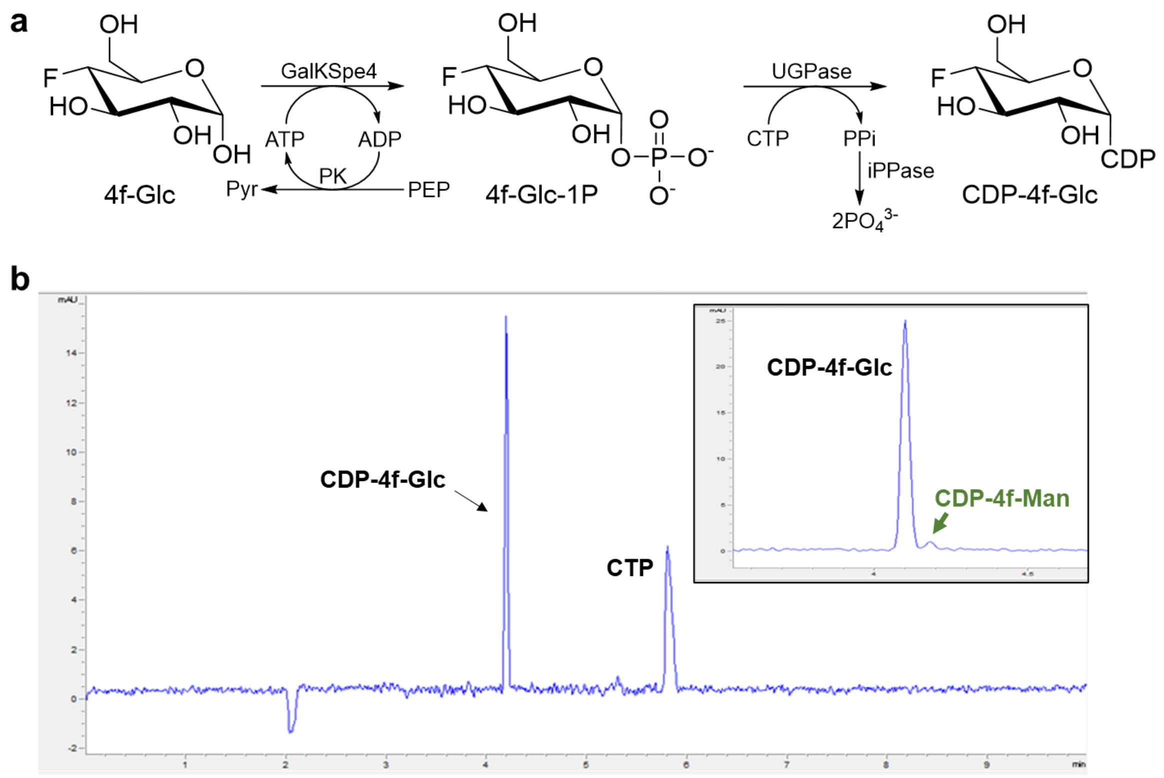

Figure S14. Synthesis route to CDP-4-deoxy-4-fluoro-α-D-glucose (CDP-4f-Glc) and CE chromatograms of the purified compound and reaction product. (**a**) The first step involves anomeric phosphorylation of 4-deoxy-4-fluoro-D-glucose (4f-Glc) by GalKSpe4, coupled to an ATP-regeneration system using PK, yielding 4-deoxy-4-fluoro-D-glucose-1-phosphate (4f-Glc-1P) and pyruvate (Pyr). UGPase and CTP were subsequently added to catalyze the formation of CDP-4f-Glc, with iPPase facilitating pyrophosphate removal. (**b**) CE chromatogram of isolated CDP-4f-Glc and CTP (21.7 mass%) with inset showing epimerization to CDP-4-deoxy-4-fluoro-α-D-mannose (CDP-4f-Man).

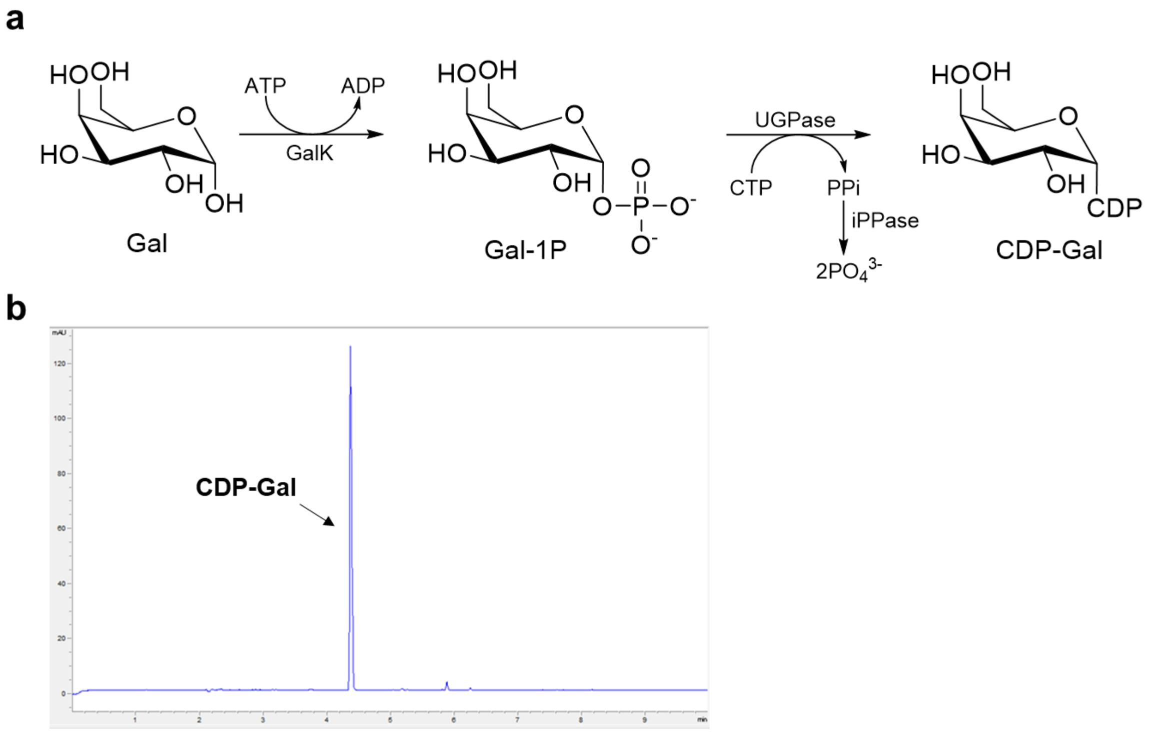

Figure S15. Enzymatic synthesis of CDP-α-D-galactose (CDP-Gal) and CE chromatogram of the isolated compound. GalK was employed to catalyze the anomeric phosphorylation of D-galactose (Gal) in the presence of ATP, yielding D-galactose-1-phosphate (Gal-1P). Subsequent addition of UGPase and CTP enabled formation of CDP-Gal. iPPase was used to promote pyrophosphate hydrolysis. (**b**) CE chromatogram of isolated CDP-Gal obtained in excellent purity ($UV_{271\ nm}$ ~99%) is shown.

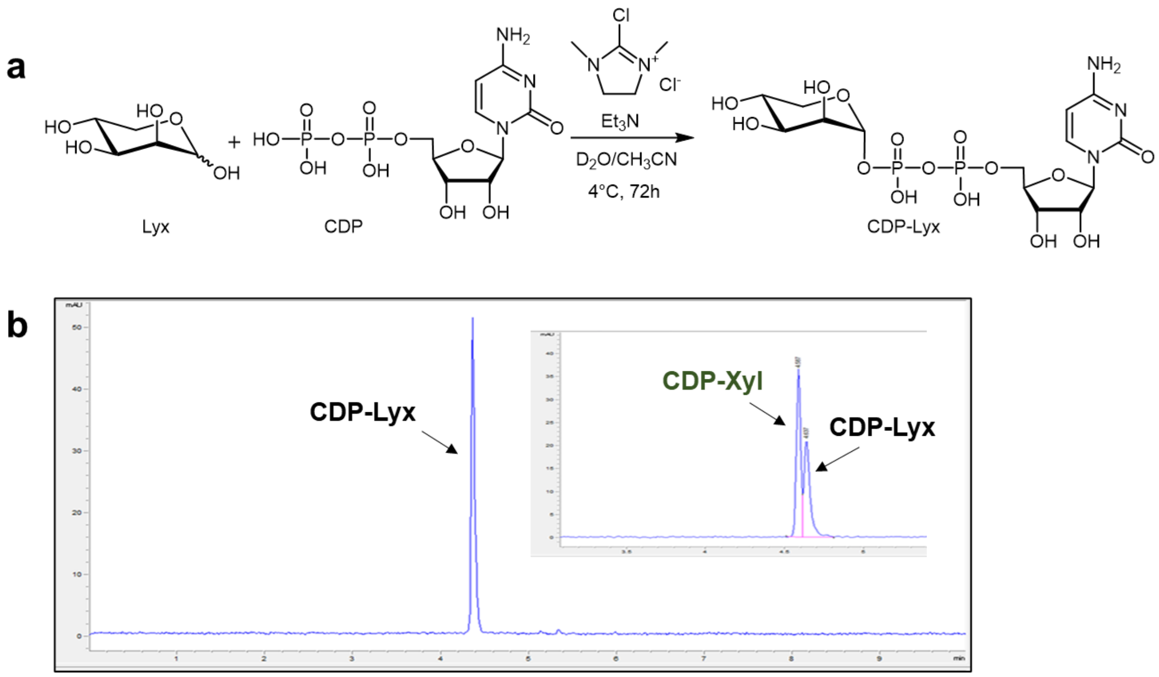

Figure S16. Single-step chemical synthesis of CDP-α-D-lyxose (CDP-Lyx). (**a**) 2-chloro-1,3-dimethylimidazolinium chloride was used to promote the condensation of CDP and D-lyxose, with triethylamine as the base (1). (**b**) CE chromatogram of isolated CDP-Lyx ($UV_{271\ nm}$ ~99%) with inset displaying the epimerization of CDP-Lyx to CDP-Xyl. Refer to discussion below for further details on synthesis.

Discussion related to **Figure S16**. Initial attempts to enzymatically phosphorylate D-lyxose and D-xylose using anomeric kinases GalKSpe4, GalK or *N*-acetylhexosamine 1-kinase (NahK; EC 2.7.1.162; from *Bifidobacterium longum*; (2)) failed. Chemical phosphorylation involving *p*-toluenesulfonohydrazide as donor, however, proved effective (3, 4). Subsequent enzymatic CMP-coupling using UGPase (5) or GDP-mannose pyrophosphorylase (GMPP; EC 2.7.7.13; from *Pyrococcus furiosus*; (6)) did not yield detectable levels of the desired sugar nucleotides. Similarly, transfer of CMP to sugar-1-phosphates in the presence of imidazole, CMP and 2-chloro-1,3-dimethylimidazolinium chloride was unsuccessful (7). The single-step chemical synthesis of sugar nucleotides as outlined in reference (1) was not demonstrated to be compatible with the formation of CDP conjugates of D-lyxose or D-xylose. However, coupling of CDP and monosaccharides proceeded, yielding products with distinct anomeric preferences: CDP-Lyx was obtained exclusively in the α-anomeric configuration, whereas CDP-Xyl was isolated as a mixture of α- and β-anomers.

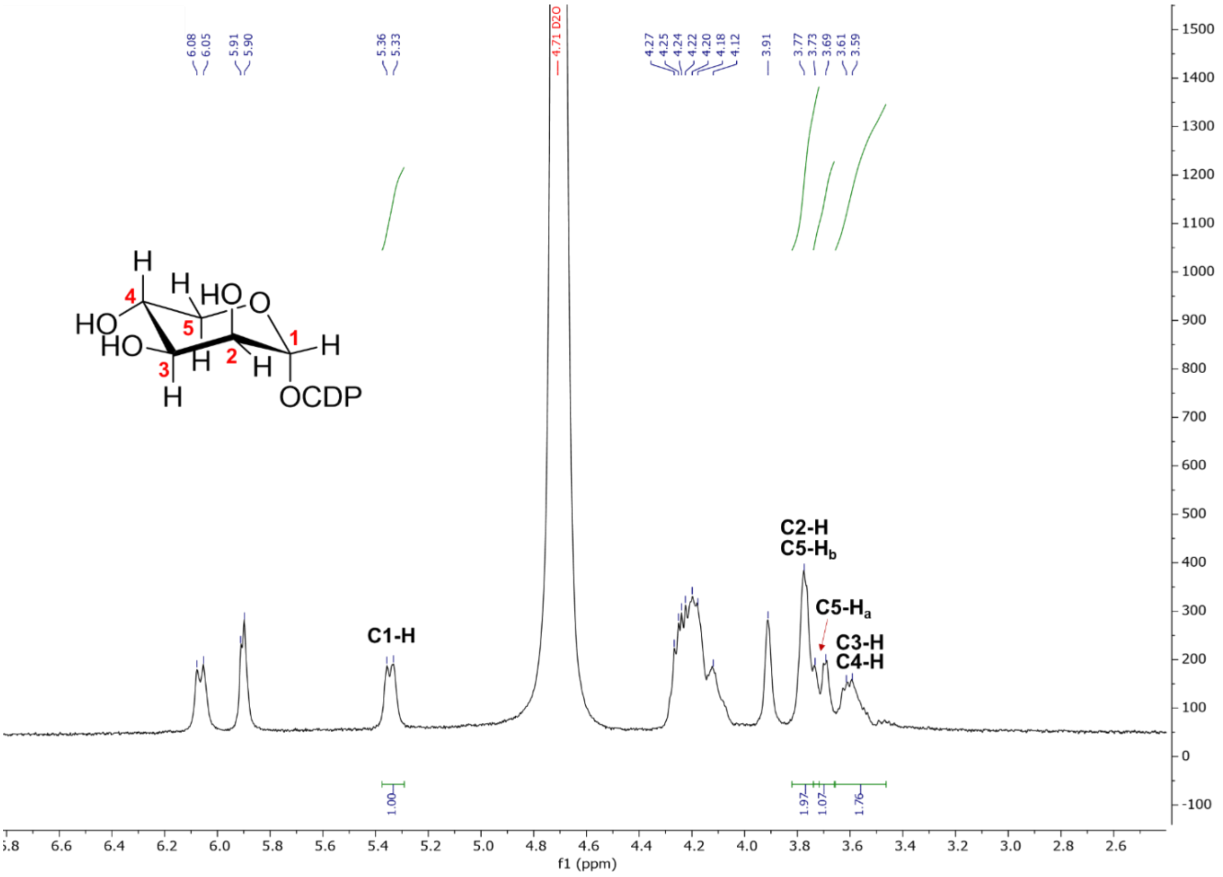

Figure S17. ^{1}H-NMR spectrum (300 MHz, D_2O) of isolated CDP-Lyx. δ 5.35 ppm (dd, 1H), 3.77 ppm (m, 2H), 3.71 ppm (dd, 1H), 3.60 ppm (m, 2H).

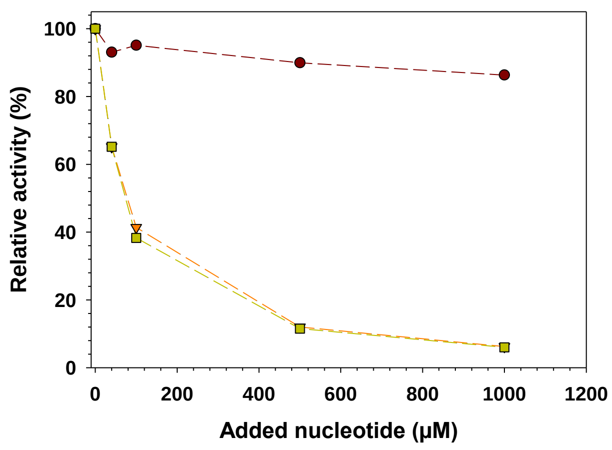

Figure S18. Inhibition of *Ta*TyvE enzyme activity by CMP, CDP and CTP. Reactions containing CDP-Glc (1.0 mM) and TaTyvE (0.5 mg mL^{-1}; 12.7 µM) were carried out in 100 mM MOPS buffer (pH 7.5) for 10 min in the presence of increasing concentrations (0–1000 µM) of CMP, CDP or CTP. Enzyme activity in the absence of added nucleotides was defined as 100% reference activity. Symbol coding: CMP (red circle), CDP (orange triangle), CTP (dark yellow square).

References

1. Miyagawa, A., Toyama, S., Ohmura, I., Miyazaki, S., Kamiya, T., and Yamamura, H. (2020) One-Step Synthesis of Sugar Nucleotides. *J. Org. Chem.* **85**, 15645–15651

2. Nishimoto, M., and Kitaoka, M. (2007) Identification of N-acetylhexosamine 1-kinase in the complete lacto-N-biose I/galacto-N-biose metabolic pathway in *Bifidobacterium longum*. *Appl. Environ. Microbiol.* **73**, 6444–6449

3. Edgar, L. J. G., Dasgupta, S., and Nitz, M. (2012) Protecting-group-free synthesis of glycosyl 1-phosphates. *Org. Lett.* **14**, 4226–4229

4. Gudmundsdottir, A. V., and Nitz, M. (2008) Protecting group free glycosidations using p-toluenesulfonohydrazide donors. *Org. Lett.* **10**, 3461–3463

5. Kotake, T., Yamaguchi, D., Ohzono, H., Hojo, S., Kaneko, S., Ishida, H. K., and Tsumuraya, Y. (2004) UDP-sugar pyrophosphorylase with broad substrate specificity toward various monosaccharide 1-phosphates from pea sprouts. *J. Biol. Chem.* **279**, 45728–45736

6. Mizanur, R. M., and Pohl, N. L. B. B. (2009) Phosphomannose isomerase/GDP-mannose pyrophosphorylase from *Pyrococcus furiosus*: a thermostable biocatalyst for the synthesis of guanidinediphosphate-activated and mannose-containing sugar nucleotides. *Org. Biomol. Chem.* **7**, 2135

7. Tanaka, H., Yoshimura, Y., and Hindsgaul, O. (2013) A simple chemical synthesis of sugar nucleoside diphosphates in water. *Curr. Protoc. Nucleic Acid Chem.* **13.12**, 1–10
